# Diagnostic accuracy of artificial intelligence versus 263 pediatric clinicians for childhood exanthems

**DOI:** 10.1007/s00431-026-07044-9

**Published:** 2026-05-08

**Authors:** Mustafa Gençeli, Özge Metin Akcan, Gonca Başak Soran, Abdulkerim Çokbiçer, Uğur Saraç, Talha Üstüntaş, Mehtap Yücel, Methiye Doğan, Ezgi Yılık Kömür, Sipil Gençeli, Hatice Yılmaz Dağlı, Memduha Sarı, Ahmet Osman Kılıç, Süleyman Şahin, Abdullah Akkuş

**Affiliations:** 1 Faculty of Medicine, Departments of Pediatric Infectious Diseases, Necmettin Erbakan University, Konya, Turkey; 2 Faculty of Medicine, Departments of Pediatrics, Necmettin Erbakan University, Konya, Turkey; 3 Faculty of Medicine, Department of Public Health, Necmettin Erbakan University, Konya, Turkey; 4 Faculty of Medicine, Departments of Pediatrics, Selçuk University, Konya, Turkey; 5 Faculty of Medicine, Departments of Pediatrics, Akdeniz University, Antalya, Turkey; 6 Departments of Pediatrics, University of Health Sciences Antalya Training and Research Hospital, Antalya, Turkey

**Keywords:** Artificial Intelligence, Diagnosise exanthematous diseases

## Abstract

Pediatric exanthematous diseases pose diagnostic challenges because clinical presentations overlap. To determine whether current artificial intelligence (AI) models achieve diagnostic accuracy within or above the performance distribution of pediatric residents and specialists for common rash-associated diseases. Participants and AI models were evaluated against definitive diagnoses confirmed by clinical features, laboratory findings, and consensus of two pediatric infectious disease specialists. A volunteer sample of 263 pediatric clinicians: 107 residents (years 1 through 4) and 156 specialists. Each clinician completed a blinded multiple-choice questionnaire with a clinical photograph and accompanying clinical data per case. The same cases were presented to three AI models: ChatGPT, Gemini, and Copilot. Among 263 clinicians (107 residents, 156 specialists), specialists scored higher than residents (median, 46 [IQR, 42—50] vs 41 [IQR, 36—46]; P < .001; r = 0.32). ChatGPT correctly diagnosed 53 of 61 cases (86.9%), Gemini 50 (82.0%), and Copilot 44 (72.1%). Both ChatGPT and Gemini exceeded the upper bound of the specialist population median 95% CI (47.17). All three AI models scored above the resident 95% CI upper bound (42.76). Disease-level accuracy ranged from 0% (insect bites, all models) to 100% (9 conditions, all models). Fourth-year residents scored higher than first- and second-year residents (*P* = .001; ε^2^ = 0.13).

*Conclusions*: AI models given clinical data alongside images matched or exceeded specialist-level performance for pediatric exanthems. Accuracy varied by disease; failures clustered in conditions that require contextual reasoning. Physician oversight remains necessary where AI accuracy is lowest.

**Table:** 

**What is Known** • *Childhood exanthematous diseases pose significant diagnostic challenges due to their overlapping clinical presentations.* • *Artificial intelligence models are becoming increasingly proficient in accurately diagnosing these conditions.*
**What is New** • *In this diagnostic accuracy study of 61 cases evaluated by 263 clinicians and 3 artificial intelligence models, ChatGPT (86.9%) and Gemini (82.0%) exceeded the 95% CI upper bound of the specialist population median. Disease-level accuracy ranged from 0 to 100% across models* • *Artificial intelligence models given clinical data alongside images can match or exceed specialist-level accuracy for common pediatric exanthems, but failures in context-dependent diagnoses require physician oversight*

**Supplementary Information:**

The online version contains supplementary material available at 10.1007/s00431-026-07044-9.

## Introduction

Pediatric exanthematous diseases are among the most frequent and diagnostically difficult presentations in pediatric practice. The differential diagnosis is broad: viral exanthems, bacterial infections, drug eruptions, and immune-mediated conditions share overlapping features at the bedside. Time-sensitive diagnoses such as meningococcemia, Kawasaki disease, and Stevens-Johnson syndrome carry serious consequences when recognition is delayed [1]. Diagnostic accuracy varies with clinical experience, yet few studies have quantified this variation across training levels.

Artificial intelligence (AI)-based tools have attracted interest as clinical decision support systems in pediatric dermatology [2]. Image analysis algorithms can aid dermatological assessment [3], but most were trained on adult datasets. Pediatric skin lesions differ from adult presentations in morphology, distribution, and clinical context, and models not calibrated to these differences lose accuracy [4]. For specific rash-associated conditions, task-trained machine learning classifiers perform well. A meta-analysis of Kawasaki disease models reported pooled accuracies exceeding 89% across external validation sites [5], and interpretable algorithms built on routine laboratory features have reached area under the curve values of 0.97 to 0.98 [6, 7]. These disease-specific tools address one condition at a time, however, and cannot generalize across the full spectrum of exanthematous diseases.

General-purpose large language models (LLMs) take a different approach. They accept unstructured clinical text and reason across multiple conditions simultaneously, which makes them candidates for broader diagnostic support. Their accuracy in pediatrics has improved rapidly but remains inconsistent. ChatGPT version 3.5 failed to identify the correct diagnosis in 83 of 100 pediatric case challenges in a previous study [8]. In pediatric dermatology, human specialists outperformed ChatGPT 3.5 on board-type and case-based questions, though version 4.0 achieved comparable accuracy [9]. Across 22 dermatological vignettes evaluated by expert raters, Copilot scored highest (approximately 90% accuracy) while ChatGPT 3.5 and Gemini clustered around 80% [10]. Our group previously showed that AI diagnostic accuracy depends heavily on input data: accuracy dropped to 30.6% when models received images alone but rose to 86.9% for ChatGPT when clinical findings accompanied images [11]. A systematic review of LLM evaluations in healthcare identified a persistent methodological limitation: most studies compare AI against panels of 1 to 5 clinicians, which prevents inference about where model performance falls within the broader distribution of clinician ability [12].

Such comparisons share a structural limitation: small clinician reference groups cannot establish whether an AI model performs at the level of a specialist, a junior trainee, or an intermediate tier. Population-level positioning of AI accuracy requires a large, stratified human comparator. We therefore evaluated the diagnostic accuracy of ChatGPT, Gemini, and Microsoft Copilot against a broad cohort of pediatric residents and specialists across common rash-associated diseases.

## Methods

This comparative multiclass diagnostic classification study was conducted at Necmettin Erbakan University Faculty of Medicine between July and December 2025. We compared the diagnostic accuracy of pediatric residents, pediatric specialists, and three AI models across 61 clinical cases of rash-associated pediatric diseases.

Sixty-one pediatric patients diagnosed with exanthematous diseases at our clinic were enrolled. Definitive diagnoses were established through clinical features, laboratory findings, and, where indicated, serological evaluation, pathological examination, and consensus of two pediatric infectious disease specialists. The 61 cases spanned 23 distinct conditions (eTable 1 in Supplement 1). The study population represented Fitzpatrick skin phototypes II through IV; no patients with phototypes V or VI were included. The patients' skin types were not provided to the AI ​​models or clinicians.

For each case, one clinical photograph and accompanying clinical information were formatted as a multiple-choice question on the Google Forms platform by two pediatric infectious disease specialists. Each question presented the clinical scenario and image with several diagnostic options, and participants selected the single most likely diagnosis. Detailed information regarding these cases and questions is provided in Supplement 2.

Pediatric residents (*n* = 107) and pediatric specialists (*n* = 156) completed the questionnaire on a voluntary basis. Residents were distributed across four training years: first year (*n* = 30), second year (*n* = 17), third year (*n* = 26), and fourth year (*n* = 34). All participants were blinded to definitive diagnoses. The same questionnaire and answer key were applied to all human participants and AI models.

The paid versions of three AI models were tested: ChatGPT Plus (GPT-5; OpenAI), Gemini (Gemini 3 Pro; Google), and Microsoft 365 Copilot (Microsoft). Models were accessed through their respective web interfaces in late 2025. Each clinical scenario, including the case image and clinical information, was presented in a new session to prevent information carryover between cases. AI-generated responses were recorded and scored against the same answer key used for human participants. Each model was tested once; run-to-run variability was not assessed.

Distribution normality was evaluated via the Kolmogorov–Smirnov test and visual inspection (histograms, Q-Q plots). Because scores were not normally distributed, results are reported as median and interquartile range (IQR). Resident-specialist comparisons used the Mann–Whitney *U* test with effect size r =|Z|/√N (0.10 small, 0.30 medium, ≥ 0.50 large). Training-year comparisons among residents employed the Kruskal–Wallis test with Dunn-Bonferroni post hoc correction; effect size was epsilon-squared (ε^2^ = [H − k + 1]/[n − k]; ≈0.01 small, ≈0.06 medium, ≥ 0.14 large).

AI model accuracy was coded as correct or incorrect per case. The Cochran Q test compared diagnostic rates across models, with McNemar tests (Bonferroni-corrected threshold *P* < 0.016) for pairwise comparisons. To position AI scores against human performance distributions, we computed 95% CIs for population medians using the Maritz-Jarrett method [15] with the formula: median ± 1.82 × IQR/√N, adapted from the McGill coefficient for 95% confidence [16]. An AI score falling outside the 95% CI indicated that model performance diverged from the expected population median of the corresponding clinician group. Because AI models produced single observations, they were excluded from inferential tests against human groups; comparisons are reported descriptively.

Disease-level subanalyses were performed via rank-based tests (Mann–Whitney *U*, Kruskal–Wallis) rather than CI-based comparisons because floor and ceiling effects in diseases with few cases (1 to 3 questions) collapsed CIs to point estimates. All analyses were conducted in IBM SPSS Statistics version 18.0 (IBM Corp). Two-sided *P* < 0.05 defined statistical significance.

The study was approved by the Ethics Committee of Necmettin Erbakan University Faculty of Medicine (Decision No. 2026/6265) and all study protocols adhered to the principles of the Declaration of Helsinki. Written informed consent was obtained from parents or legal guardians of all patients.

## Results

**Fig. 1:**
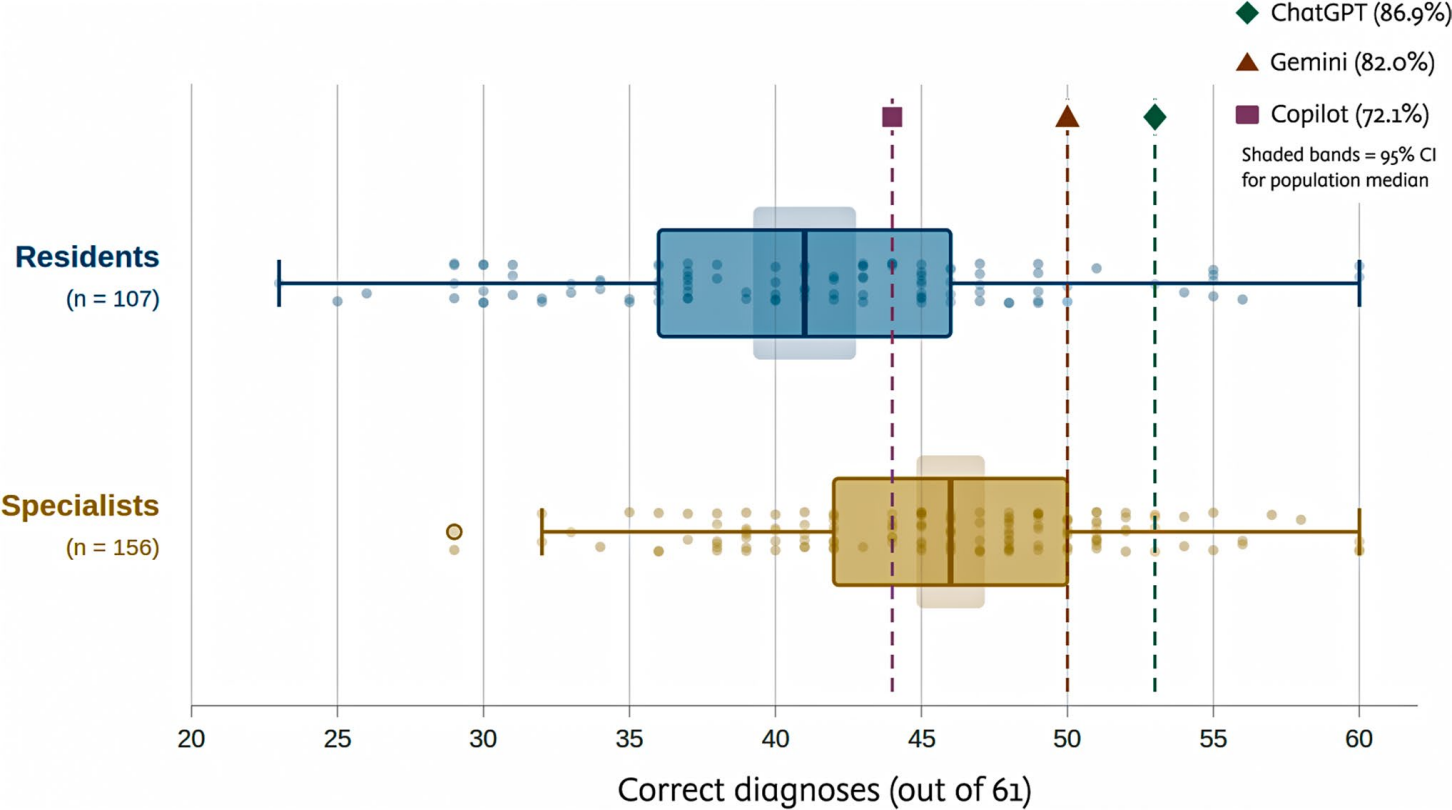
Evaluation of overall diagnostic accuracy for clinicians and artificial intelligence models

A total of 263 clinicians participated: 107 residents (40.7%) and 156 specialists (59.3%). Participant characteristics and overall diagnostic accuracy are shown in Fig. 1. Specialists achieved a higher total diagnostic score than residents (median 46 [IQR, 42—50] vs 41 [IQR, 36—46]; P < 0.001; r = 0.32). Disease-level comparisons between residents and specialists are presented in Supplement 1 (eTable 2).

Among AI models, ChatGPT achieved 53 of 61 correct diagnoses (86.9%), Gemini achieved 50 (82.0%), and Copilot achieved 44 (72.1%). The Cochran Q test identified a significant difference across models (*P* = 0.030). Pairwise McNemar tests showed that ChatGPT outperformed Copilot (*P* = 0.022) while no significant differences emerged between ChatGPT and Gemini (*P* = 0.508) or between Gemini and Copilot (*P* = 0.180). When AI scores were evaluated against the 95% CI for the specialist population median (44.83—47.17), ChatGPT (53) and Gemini (50) exceeded the upper bound, indicating performance above the expected specialist median. Copilot (44) fell marginally below the lower bound. All three AI models exceeded the upper bound of the resident population median 95% CI (39.24—42.76) (Table 1).

**Table 1:** AI Model comparisons and positioning relative to clinician performance distributions

Panel A: Pairwise AI Model Comparisons				
AI Model	Correct, No. (%)	Incorrect, No. (%)		
ChatGPT	53 (86.9)	8 (13.1)		
Gemini	50 (82.0)	11 (18.0)		
Copilot	44 (72.1)	17 (27.9)		
Panel B: AI Scores Relative to Human Population Median 95% CIs				
Group/Model	Score	95% CI	vs Specialist CI	vs Resident CI
Specialists (*n* = 156)	46	44.83—47.17	*Reference*	—
Residents (*n* = 107)	41	39.24—42.76	Below CI	*Reference*
ChatGPT	53	—	Above upper bound	Above upper bound
Gemini	50	—	Above upper bound	Above upper bound
Copilot	44	—	Marginally below	Above upper bound

Cochran Q test: *P* =.030

Post hoc (McNemar, Bonferroni-corrected threshold *P* <.016): ChatGPT vs Copilot: *P* =.022; ChatGPT vs Gemini: *P* =.508; Gemini vs Copilot: *P* =.180

CIs computed as median ± 1.82 × IQR/√N (Maritz-Jarrett method). AI models tested once; CIs not computed for single observations

**Fig. 2:**
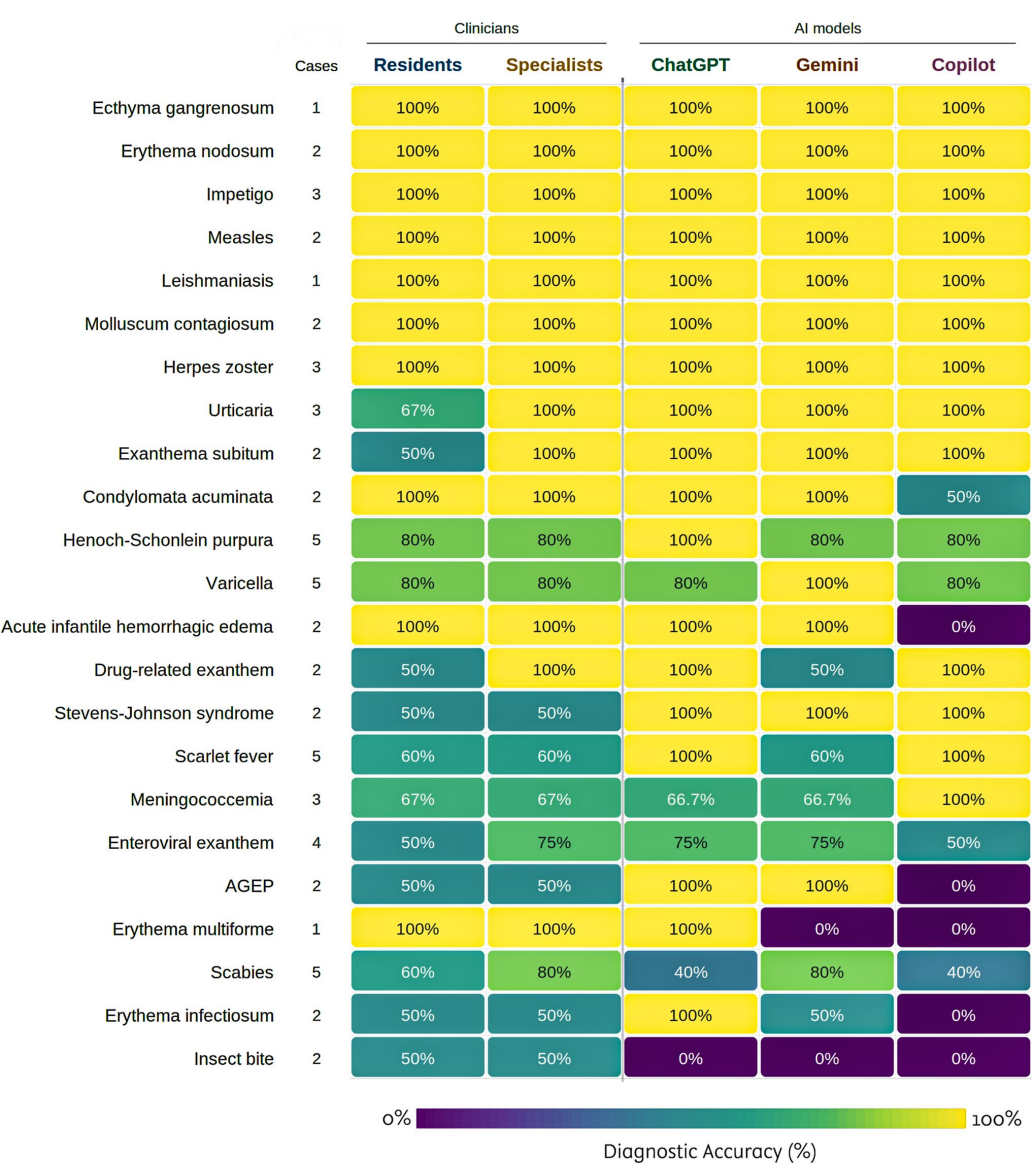
Evaluation of diagnostic accuracy of clinicians and artificial intelligence models across various clinical settings

Disease-level accuracy varied widely across models and conditions (Fig. 2). All three models correctly diagnosed impetigo, erythema nodosum, exanthema subitum, measles, leishmaniasis, urticaria, molluscum contagiosum, Stevens-Johnson syndrome, and herpes zoster (100% accuracy per model). All three models failed to identify any insect bite case (0% accuracy). Scabies accuracy was 40% for both ChatGPT and Copilot; Gemini reached 80%. Parvovirus B19 accuracy ranged from 0% (Copilot) to 100% (ChatGPT). Copilot failed all cases of acute infantile hemorrhagic edema and acute generalized exanthematous pustulosis (0%), while both ChatGPT and Gemini identified all cases (100%).

**Fig. 3:**
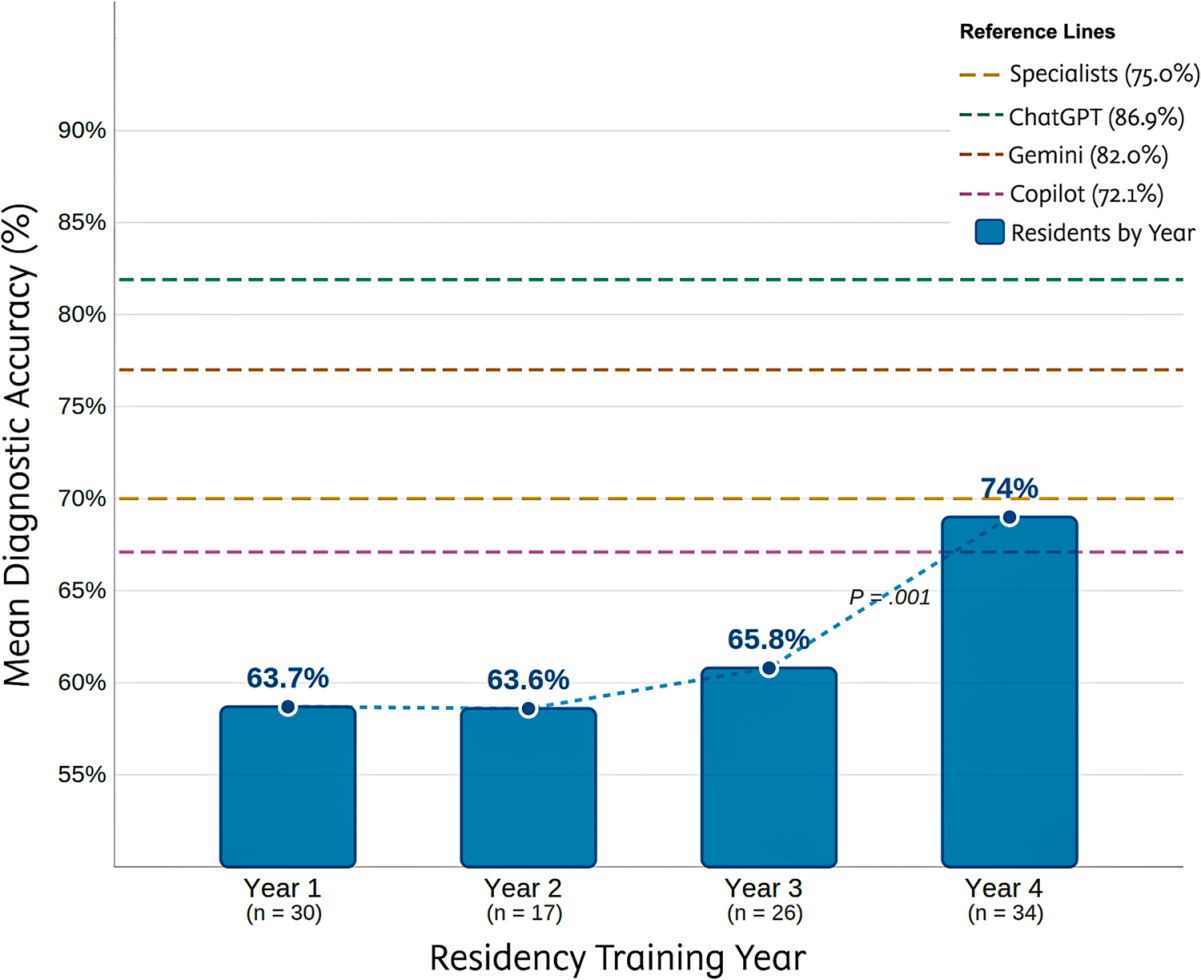
Correlation between clinical experience (training year) and diagnostic performance

Among residents, diagnostic accuracy increased with training year (Table 2, Fig. 3). Fourth-year residents scored higher than first- and second-year residents on total accuracy (median 45 vs 40 and 37; *P* = 0.001; ε^2^ = 0.13). Three diseases drove this separation: enteroviral disease (*P* = 0.001; post hoc: years 1, 2, and 3 < year 4; ε^2^ = 0.13), Henoch-Schönlein purpura (*P* = 0.002; year 1 < years 3 and 4; ε^2^ = 0.11), and meningococcemia (*P* = 0.001; year 1 < years 2 and 4; ε^2^ = 0.13). Comprehensive disease-level data according to training year are presented in eTable 3 (Supplement 1).

**Table 2:** Resident diagnostic accuracy by training year: total score and diseases with significant differences

Variable	Year 1 (*n* = 30)	Year 2 (*n* = 17)	Year 3 (*n* = 26)	Year 4 (*n* = 34)	*P* Value	Post hoc	ε^2^
Total score	40 (33—45)	37 (32—40.5)	41 (34—46)	45 (41—49)	.001	Yr 1,2 < 4	0.13
Enteroviral disease	2 (1—3)	2 (1—2)	2 (1—3)	3 (2—4)	.001	Yr 1,2,3 < 4	0.13
Henoch-Schönlein purpura	4 (3—4.2)	4 (3—5)	5 (4—5)	5 (4—5)	.002	Yr 1 < 3,4	0.11
Meningococcemia	1 (1—2)	3 (2—3)	2 (1—2)	2 (2—3)	.001	Yr 1 < 2,4	0.13

Values are median (IQR) of correct diagnoses. Kruskal–Wallis test. Post hoc: Dunn-Bonferroni correction; only significant comparisons shown

ε^2^ ≈ 0.01 small, ≈ 0.06 medium, ≥ 0.14 large. Full disease-level data: eTable 3 (Supplement 1)

## Discussion

ChatGPT achieved the highest diagnostic accuracy among the AI models (86.9%), followed by Gemini (82.0%) and Copilot (72.1%). Both ChatGPT and Gemini scored above the upper bound of the 95% confidence interval for the specialist population median (47.17 of 61), placing their performance outside the expected range of specialist accuracy. Copilot fell marginally below the specialist confidence interval lower bound but exceeded the resident upper bound. Among human participants, specialists outperformed residents (median 46 vs 41; *P* < 0.001; r = 0.32), and fourth-year residents scored higher than first- and second-year residents (*P* = 0.001; ε^2^ = 0.13). These results reflect rapid improvement over earlier LLM benchmarks. Barile et al. [8] reported an 83% diagnostic error rate when ChatGPT 3.5 was tested on 100 pediatric case challenges drawn from published literature. Our study, using GPT-5 on real clinical cases with accompanying images and clinical data, found the opposite pattern: an 87% correct diagnosis rate. The difference likely reflects both model evolution and the provision of structured clinical context alongside images. Our group’s prior work established that clinical context is the decisive variable. Without it, the same models achieved accuracy as low as 30.6% [11]. Huang et al. [9] found that pediatric dermatologists outperformed ChatGPT 3.5 but not version 4.0 on text-based questions. Podder et al. [10] reported that Copilot outperformed ChatGPT 3.5 and Gemini in their cohort. We observed the reverse ranking: ChatGPT outperformed Copilot (*P* = 0.022), while the difference between ChatGPT and Gemini was not significant. Because each study tested different model versions, direct comparison is limited. What remains consistent across studies is that model identity matters less than model generation: newer iterations outperform older ones regardless of brand.

Our disease-level analyses revealed patterns that aggregate accuracy scores obscure. All three models correctly identified morphologically distinct conditions with well-defined clinical features: impetigo, urticaria, herpes zoster, molluscum contagiosum, measles, and Stevens-Johnson syndrome were diagnosed at or near 100% accuracy by each model. Conditions that require integrating subtle clinical history with nonspecific skin findings proved harder. All three models failed every insect bite case (0% accuracy across models). Scabies accuracy was 40% for ChatGPT and Copilot (Gemini reached 80%). Parvovirus B19 showed a steep gradient: ChatGPT identified both cases, Gemini identified one, Copilot identified neither. These failures are clinically relevant. Insect bites are benign but trigger unnecessary workups when misclassified; scabies requires specific treatment that delays when missed. The pattern suggests that LLMs perform best when a diagnosis maps cleanly onto textbook descriptions and perform worst when the diagnosis depends on contextual reasoning about exposure history, distribution patterns, or epidemiologic clues. Mathes et al. [13] identified analogous failures in allergology, where ChatGPT produced critical errors in pediatric food allergen counseling. Su et al. [14] showed that LLM recommendation quality declines as clinical scenarios grow more complex. Both findings align with the disease-level variation we observed.

The training-year gradient among residents deserves attention for a different reason. Fourth-year residents (median 45; mean 74.0% accuracy) approached the specialist median (46), while first- and second-year residents scored substantially lower (medians 40 and 37). The diseases driving this separation were enteroviral exanthems, Henoch-Schönlein purpura, and meningococcemia. These conditions demand integration of systemic features with cutaneous findings. The implication for medical education is straightforward: clinical pattern recognition for rash-associated diseases improves with cumulative exposure, and the steepest gains occur in the final year of training. That AI models exceeded even the specialist population median raises the question of whether these tools could serve as calibration benchmarks during residency training.

Our study has certain limitations. Each AI model was tested once, so run-to-run variability could not be assessed; AI scores should be interpreted as point estimates. The study population included only Fitzpatrick skin phototypes II through IV, and performance on darker skin tones remains untested. Patients' skin types were unknown to artificial intelligence models and clinicians. The multiple-choice format may overestimate accuracy relative to open-ended differential diagnosis, which better approximates clinical reasoning. Cases were drawn from a single Turkish academic center, and generalizability to other populations and clinical settings requires further study. Human participants volunteered to complete the survey, introducing potential selection bias toward more engaged clinicians. AI models were queried at a single time point in late 2025; subsequent model updates could change performance in either direction.

Despite these constraints, the large clinician sample (*n* = 263) stratified by training level allows population-level inference that prior studies with 1 to 5 human comparators could not achieve. Current-generation LLMs, when provided with clinical data alongside images, match or exceed the expected diagnostic accuracy of pediatric specialists for common exanthematous diseases. Translating this performance into clinical benefit is a separate problem. It will require prospective validation across diverse skin phototypes, standardized prompting protocols, and integration into workflows that preserve physician oversight for the conditions where these models still fail. The disease-level variation we observed suggests that deployment should be selective: AI-assisted triage may be appropriate for morphologically distinct conditions, but context-dependent diagnoses remain the province of clinical judgment.

## Supplementary Information

Below is the link to the electronic supplementary material.

Supplementary file1 (DOCX 25 KB)

Supplementary file2 (DOCX 34367 KB)

## Data Availability

No datasets were generated or analysed during the current study.

## Abbreviations

AI: Artificial intelligence
LLM: Large language model

## References

[1] Sarkar R, Yadav A, Maheshwari A (2024) Fever with rash in a child: revisited. Indian J Dermatol 69(3):282. 10.4103/ijd.ijd_913_23 39119300 10.4103/ijd.ijd_913_23 PMC11305486

[2] Zama D, Borghesi A, Ranieri A, Manieri E, Pierantoni L, Andreozzi L, Dondi A, Neri I, Lanari M, Calegari R (2024) Perspectives and challenges of telemedicine and artificial intelligence in pediatric dermatology. Children Basel 11(11):1401. 10.3390/children11111401 39594976 10.3390/children11111401 PMC11592520

[3] Li Z, Koban KC, Schenck TL, Giunta RE, Li Q, Sun Y (2022) Artificial intelligence in dermatology image analysis: current developments and future trends. J Clin Med 11(22):6826. 10.3390/jcm11226826 36431301 10.3390/jcm11226826 PMC9693628

[4] Mehta PP, Sun M, Betz-Stablein B, Halpern A, Soyer HP, Weber J, Kose K, Rotemberg V (2023) Improving artificial intelligence-based diagnosis on pediatric skin lesions. J Invest Dermatol 143(8):1423-1429.e1. 10.1016/j.jid.2022.08.058 36804150 10.1016/j.jid.2022.08.058 PMC10431965

[5] Zhu J, Yang F, Wang Y, Wang Z, Xiao Y, Wang L, Sun L (2024) Accuracy of machine learning in discriminating Kawasaki disease and other febrile illnesses: systematic review and meta-analysis. JMIR Med Inform 12:e57641. 10.2196/57641 10.2196/57641 PMC11612596 39556821

[6] Duan Y, Wang R, Huang Z, Chen H, Tang M, Zhou J, Hu Z, Hu W, Chen Z, Qian Q, Wang H (2024) Intelligent diagnosis of Kawasaki disease from real-world data using interpretable machine learning models. Hellenic J Cardiol 81:38–48. 10.1016/j.hjc.2024.08.003 39128707 10.1016/j.hjc.2024.08.003

[7] Duan M, Geng Z, Gao L, Zhao Y, Li Z, Chen L, Kuosmanen P, Qi G, Gong F, Yu G (2025) An interpretable machine learning-assisted diagnostic model for Kawasaki disease in children. Sci Rep 15(1):2277. 10.1038/s41598-025-92277-1 40050685 10.1038/s41598-025-92277-1 PMC11885592

[8] Barile J, Margolis A, Cason G, Kim R, Kalash S, Tchaconas A, Milanaik R (2024) Diagnostic accuracy of a large language model in pediatric case studies. JAMA Pediatr 178(3):313–315. 10.1001/jamapediatrics.2023.5750 38165685 10.1001/jamapediatrics.2023.5750 PMC10762631

[9] Huang CY, Zhang E, Caussade MC, Brown T, Stockton Hogrogian G, Yan AC (2024) Pediatric dermatologists versus AI bots: evaluating the medical knowledge and diagnostic capabilities of ChatGPT. Pediatr Dermatol 41(5):831–834. 10.1111/pde.15649 38721744 10.1111/pde.15649

[10] Podder I, Pipil N, Dhabal A, Mondal S, Pienyii V, Mondal H (2024) Evaluation of artificial intelligence-based chatbot responses to common dermatological queries. Jordan Med J 58(2). 10.35516/jmj.v58i2.2960

[11] Gençeli M, Soran GB, Metin Akcan Ö, Yücel M, Üstüntaş T, Saraç U, Çokbiçer A, Kaygusuz Aydemir B, Erdoğan C, Kaba E, Şahin S (2025) Diagnostic accuracy of artificial intelligence models in childhood exanthematous diseases: a comparative analysis against clinical diagnosis. Eur J Pediatr 185(1):33. 10.1007/s00431-025-06693-6 41428260 10.1007/s00431-025-06693-6

[12] Bedi S, Liu Y, Orr-Ewing L, Dash D, Koyejo S, Callahan A, Fries JA, Wornow M, Swaminathan A, Lehmann LS, Hong HJ, Kashyap M, Chaurasia AR, Shah NR, Singh K, Tazbaz T, Milstein A, Pfeffer MA, Shah NH (2025) Testing and evaluation of health care applications of large language models: systematic review. JAMA 333(4):319–328. 10.1001/jama.2024.21700 39405325 10.1001/jama.2024.21700 PMC11480901

[13] Mathes S, Seurig S, Bluhme F, Beyer K, Heizmann F, Wagner M, Neugärtner I, Biedermann T, Darsow U (2025) ChatGPT performance on 120 interdisciplinary allergology questions: systematic evaluation with clinical error impact assessment for critical erroneous AI-guided chatbot advice. J Allergy Clin Immunol Pract. 10.1016/j.jaip.2025.03.030 40157421 10.1016/j.jaip.2025.03.030

[14] Su J, Yang X, Li X, Chen J, Jiang C, Wang Y, Zhuang L, Li H (2025) Evaluating large language models for accuracy and completeness of vitiligo patient education: a comparative analysis. Clin Cosmet Investig Dermatol 18:457–466. 10.2147/CCID.S552979 10.2147/CCID.S552979 PMC12558161 41158792

[15] Maritz JS, Jarrett RG (1978) A note on estimating the variance of the sample median. J Am Stat Assoc 73(361):194–196. 10.2307/2286670

[16] McGill R, Tukey JW, Larsen WA (1978) Variations of box plots. The American Statistician 32(1):12–16. 10.1080/00031305.1978.10479236

