# Supplementary material for: Diagnostic accuracy of artificial intelligence versus 263 pediatric clinicians for childhood exanthems
Source: Eur J Pediatr. 2026 May 8;185(6):372. doi: 10.1007/s00431-026-07044-9 (PMC13156224; doi:10.1007/s00431-026-07044-9)
Supplement: Supplementary file 1 — Supplementary file1 (DOCX 25 KB) [file 431_2026_7044_MOESM1_ESM.docx]

**Supplement 1**

**Diagnostic Accuracy of AI Versus 263 Pediatric Clinicians for Childhood Exanthems**

**eTable 1. Distribution of 23 Diseases and Case Numbers**

| **Disease** | **No. of Cases** | **Case Numbers** |
| --- | --- | --- |
| Acute infantile hemorrhagic edema | 2 | 1, 28 |
| Acute generalized exanthematous pustulosis | 2 | 2, 29 |
| Ecthyma gangrenosum | 1 | 3 |
| Enteroviral exanthem | 4 | 4, 30, 31, 57 |
| Erythema multiforme | 1 | 5 |
| Erythema nodosum | 2 | 6, 59 |
| Impetigo | 3 | 10, 39, 60 |
| Exanthema subitum (roseola) | 2 | 7, 54 |
| Henoch-Schönlein purpura | 5 | 8, 32, 51, 56, 60 |
| Drug-related exanthem | 2 | 9, 33 |
| Scarlet fever | 5 | 11, 16, 23, 26, 37 |
| Measles | 2 | 12, 36 |
| Leishmaniasis | 1 | 13 |
| Meningococcemia | 3 | 14, 19, 40 |
| Insect bite | 2 | 15, 42 |
| Urticaria | 3 | 17, 22, 45 |
| Molluscum contagiosum | 2 | 18, 53 |
| Stevens-Johnson syndrome | 2 | 20, 55 |
| Scabies | 5 | 21, 44, 46, 49, 58 |
| Varicella | 5 | 24, 34, 38, 43, 48 |
| Condylomata acuminata | 2 | 25, 50 |
| Herpes zoster | 3 | 27, 35, 47 |
| Erythema infectiosum (parvovirus B19) | 2 | 52, 61 |
| **Total** | **61** |  |

Case numbers correspond to the clinical case vignettes in the eAppendix (uploaded separately).

**eTable 2. Diagnostic Accuracy by Disease: Residents vs Specialists**

| **Disease** | **Cases** | **All (n=263)** | **Residents (n=107)** | **Specialists (n=156)** | **P** | **r** |
| --- | --- | --- | --- | --- | --- | --- |
| **Total score** | 61 | 46 (41-50) | 41 (36-46) | 46 (42-50) | **<.001** | 0.32 |
| Acute infantile hemorrhagic edema | 2 | 2 (1-2) | 2 (1-2) | 2 (1-2) | **<.001** | 0.24 |
| AGEP | 2 | 1 (1-2) | 1 (1-2) | 1 (1-1) | .304 |  |
| Ecthyma gangrenosum | 1 | 1 (0-1) | 1 (0-1) | 1 (0-1) | .863 |  |
| Enteroviral exanthem | 4 | 3 (2-3) | 2 (1-3) | 3 (2-4) | **<.001** | 0.26 |
| Erythema multiforme | 1 | 1 (0-1) | 1 (0-1) | 1 (0-1) | **.029** | 0.13 |
| Erythema nodosum | 2 | 2 (2-2) | 2 (1-2) | 2 (2-2) | **.042** | 0.13 |
| Impetigo | 3 | 3 (2-3) | 3 (2-3) | 3 (3-3) | **.029** | 0.13 |
| Exanthema subitum | 2 | 2 (1-2) | 1 (1-2) | 2 (1-2) | **<.001** | 0.32 |
| HSP | 5 | 4 (4-5) | 4 (4-5) | 4 (4-5) | .371 |  |
| Drug-related exanthem | 2 | 2 (1-2) | 1 (1-2) | 2 (1-2) | **.007** | 0.17 |
| Scarlet fever | 5 | 3 (2-4) | 3 (2-4) | 3 (2-4) | .391 |  |
| Measles | 2 | 2 (1-2) | 2 (1-2) | 2 (2-2) | **.001** | 0.21 |
| Leishmaniasis | 1 | 1 (1-1) | 1 (0-1) | 1 (1-1) | .144 |  |
| Meningococcemia | 3 | 2 (2-3) | 2 (1-3) | 2 (2-3) | **.022** | 0.14 |
| Insect bite | 2 | 1 (0-1) | 1 (0-1) | 1 (0-1) | .570 |  |
| Urticaria | 3 | 3 (2-3) | 2 (2-3) | 3 (2-3) | .577 |  |
| Molluscum contagiosum | 2 | 2 (1-2) | 2 (1-2) | 2 (1-2) | **.024** | 0.14 |
| Stevens-Johnson syndrome | 2 | 1 (1-2) | 1 (1-2) | 1 (1-2) | .669 |  |
| Scabies | 5 | 3 (2-4) | 3 (2-4) | 4 (3-4) | **<.001** | 0.28 |
| Varicella | 5 | 4 (3-5) | 4 (3-5) | 4 (3-5) | **.034** | 0.13 |
| Condylomata acuminata | 2 | 2 (1-2) | 2 (1-2) | 2 (1-2) | .185 |  |
| Herpes zoster | 3 | 3 (3-3) | 3 (3-3) | 3 (3-3) | **<.001** | 0.23 |
| Erythema infectiosum | 2 | 1 (1-1) | 1 (1-2) | 1 (1-1) | .488 |  |

*Values are median (IQR) of correct diagnoses. Mann-Whitney U test.*

*Effect size r = |Z|/√N; 0.10 small, 0.30 medium, ≥0.50 large. Bold P values indicate statistical significance (P < .05).*

*AGEP indicates acute generalized exanthematous pustulosis; HSP, Henoch-Schönlein purpura.*

**eTable 3. Resident Diagnostic Accuracy by Training Year: All 23 Diseases**

| **Disease** | **Year 1 (n=30)** | **Year 2 (n=17)** | **Year 3 (n=26)** | **Year 4 (n=34)** | **P** | **Post hoc** | **ε²** |
| --- | --- | --- | --- | --- | --- | --- | --- |
| **Total score** | 40 (33-45) | 37 (32-40.5) | 41 (34-46) | 45 (41-49) | **.001** | Yr 1,2 < 4 | 0.13 |
| Acute infantile hemorrhagic edema | 1 (0.7-2) | 1 (1-2) | 1 (0.7-2) | 2 (1-2) | .050 |  |  |
| AGEP | 1 (0-2) | 1 (1-2) | 1 (0.7-1.25) | 1 (0-2) | .631 |  |  |
| Ecthyma gangrenosum | 1 (0-1) | 0 (0-1) | 1 (0-1) | 1 (0.75-1) | .183 |  |  |
| Enteroviral exanthem | 2 (1-3) | 2 (1-2) | 2 (1-3) | 3 (2-4) | **.001** | Yr 1,2,3 < 4 | 0.13 |
| Erythema multiforme | 1 (0-1) | 1 (0-1) | 0 (0-1) | 1 (0-1) | .528 |  |  |
| Erythema nodosum | 2 (2-2) | 2 (1-2) | 2 (1-2) | 2 (1.75-2) | .697 |  |  |
| Impetigo | 3 (2-3) | 2 (1.5-3) | 3 (2-3) | 3 (2-3) | .125 |  |  |
| Exanthema subitum | 1 (0-2) | 2 (1-2) | 2 (0-2) | 1 (1-2) | .449 |  |  |
| HSP | 4 (3-4.2) | 4 (3-5) | 5 (4-5) | 5 (4-5) | **.002** | Yr 1 < 3,4 | 0.11 |
| Drug-related exanthem | 1 (1-2) | 1 (0-2) | 1 (1-2) | 1.5 (1-2) | .789 |  |  |
| Scarlet fever | 3 (2-4) | 3 (1.5-4) | 3 (2-4) | 3 (2-4) | .785 |  |  |
| Measles | 2 (1-2) | 1 (1-2) | 1.5 (0.7-2) | 2 (1-2) | .081 |  |  |
| Leishmaniasis | 1 (0-1) | 1 (0.5-1) | 1 (0-1) | 1 (1-1) | .669 |  |  |
| Meningococcemia | 1 (1-2) | 3 (2-3) | 2 (1-2) | 2 (2-3) | **.001** | Yr 1 < 2,4 | 0.13 |
| Insect bite | 1 (0-1) | 1 (0.5-1) | 1 (1-1) | 1 (0-1) | .510 |  |  |
| Urticaria | 2 (2-3) | 2 (1.5-3) | 2 (2-3) | 3 (2-3) | .122 |  |  |
| Molluscum contagiosum | 1 (0.7-2) | 1 (0-2) | 2 (1-2) | 2 (1-2) | .152 |  |  |
| Stevens-Johnson syndrome | 1 (1-1.2) | 1 (1-2) | 1 (0.7-2) | 1.5 (1-2) | .411 |  |  |
| Scabies | 3 (2-3) | 3 (2-4) | 3 (2-3) | 3 (2-4) | .196 |  |  |
| Varicella | 3.5 (3-4) | 4 (2.5-4) | 3.5 (2-5) | 4 (3-5) | .190 |  |  |
| Condylomata acuminata | 1 (1-2) | 1 (0-2) | 1 (0.7-2) | 2 (1-2) | .080 |  |  |
| Herpes zoster | 3 (2-3) | 3 (2-3) | 3 (3-3) | 3 (3-3) | .135 |  |  |
| Erythema infectiosum | 1 (1-2) | 1 (0-2) | 1 (1-1) | 1 (1-1.25) | .115 |  |  |

*Values are median (IQR) of correct diagnoses. Kruskal-Wallis test.*

*Post hoc: Dunn-Bonferroni correction; only significant comparisons shown. Bold P values indicate statistical significance (P < .05).*

*ε² ≈ 0.01 small, ≈ 0.06 medium, ≥ 0.14 large.*

*AGEP indicates acute generalized exanthematous pustulosis; HSP, Henoch-Schönlein purpura.*
