# Supplementary material for: Diagnostic accuracy of artificial intelligence versus 263 pediatric clinicians for childhood exanthems
Source: Eur J Pediatr. 2026 May 8;185(6):372. doi: 10.1007/s00431-026-07044-9 (PMC13156224; doi:10.1007/s00431-026-07044-9)
Supplement: Supplementary file 2 — Supplementary file2 (DOCX 34367 KB) [file 431_2026_7044_MOESM2_ESM.docx]

1. **An 18-month-old female patient** presented with **widespread redness on the body for the past 2 days and some skin lesions that appeared black in color**. The black-colored lesions **did not blanch on pressure**. Lesions were also present **on the auricle (ear)**. **Her feet were edematous.** There were **no additional notable findings**. **What is your diagnosis?**

**
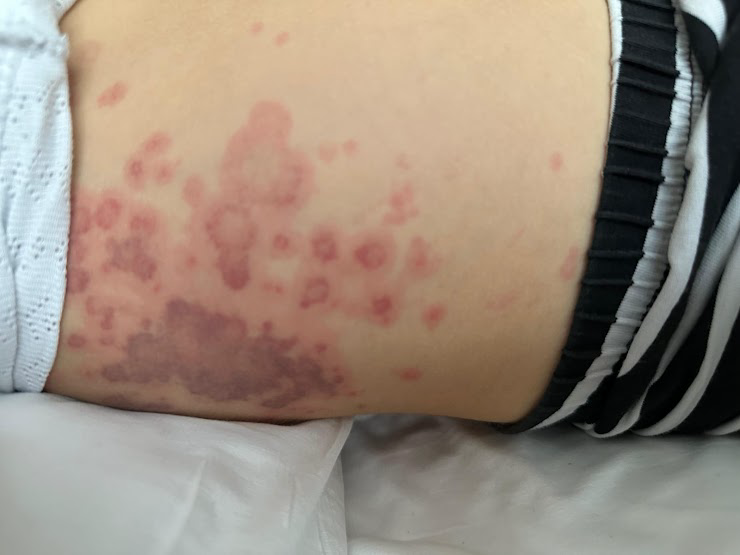
**

 **Urticaria**

 **Acute Infantile Hemorrhagic Edema (AIHE)**

 **Henoch–Schönlein Purpura (HSP)**

 **Meningococcemia**

 **Drug Rash with Eosinophilia and Systemic Symptoms (DRESS)**

1. A **7-year-old male patient** presented with **fever and rash for the past 2 days**. The **fever was persistent/resistant**. The **rash started on the neck and spread to the entire trunk**. **Small pustular lesions were present in some areas.** The **oropharynx was hyperemic**. **Other system examinations were normal. What is your diagnosis?**

**
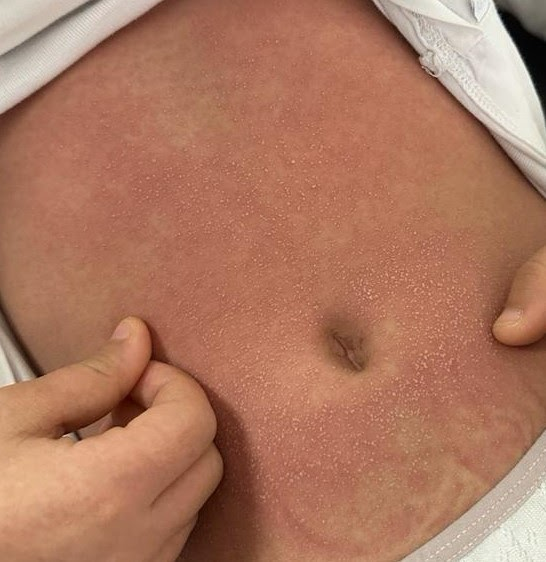
**

 **Scarlet Fever**

 **Impetigo Herpetiformis**

 **Acute Generalized Exanthematous Pustulosis (AGEP)**

 **Drug Rash with Eosinophilia and Systemic Symptoms (DRESS)**

 **Disseminated Gonococcal Infection**

1. An **8-month-old male patient** presented with **persistent fever for 9 days and ear discharge**. There were **three lesions in the gluteal region with black, depressed centers**. **Tissue loss was present.** The **patient appeared toxic** and was **malnourished**. **Leukocytosis and elevated CRP levels** were detected.**What is your diagnosis?**

**
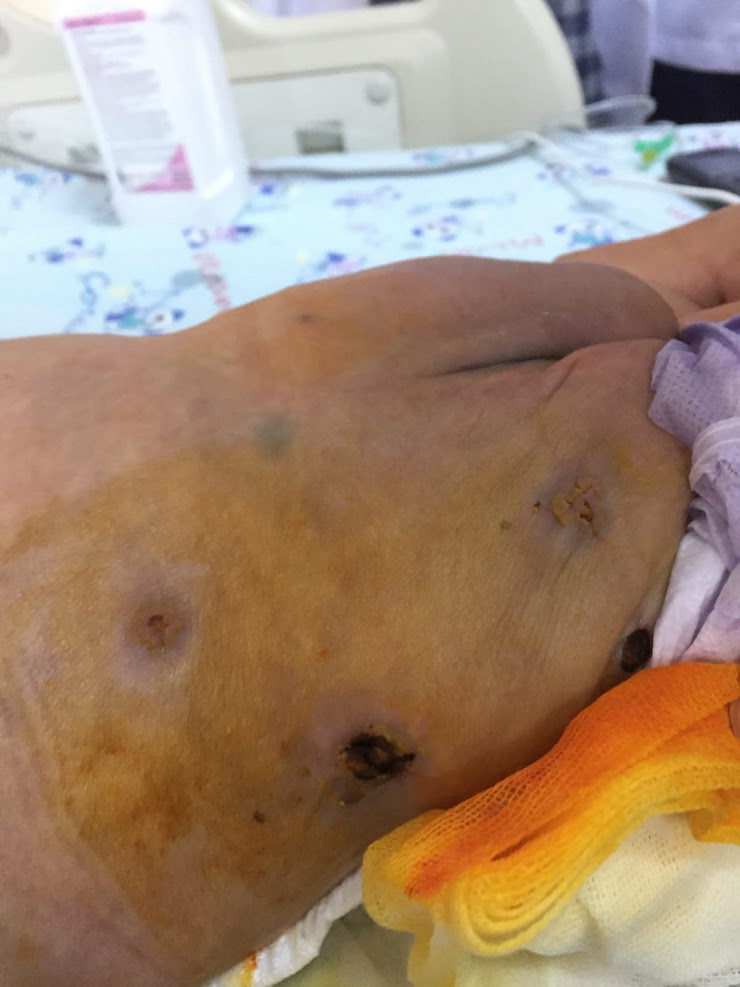
**

 **Purpura Fulminans**

 **Cutaneous Leishmaniasis**

 **Impetigo**

 **Ecthyma Gangrenosum**

 **Disseminated Gonococcal Infection**

1. A **16-month-old male patient** presented with a **rash for the past 3 days**, which **started on the hands and feet and spread to the arms and legs**. He had also had **fever for the past 2 days**. **Ten days earlier, he had contact with a patient who had a similar rash with lesions on the hands, feet, and mouth.** **What is your diagnosis?**


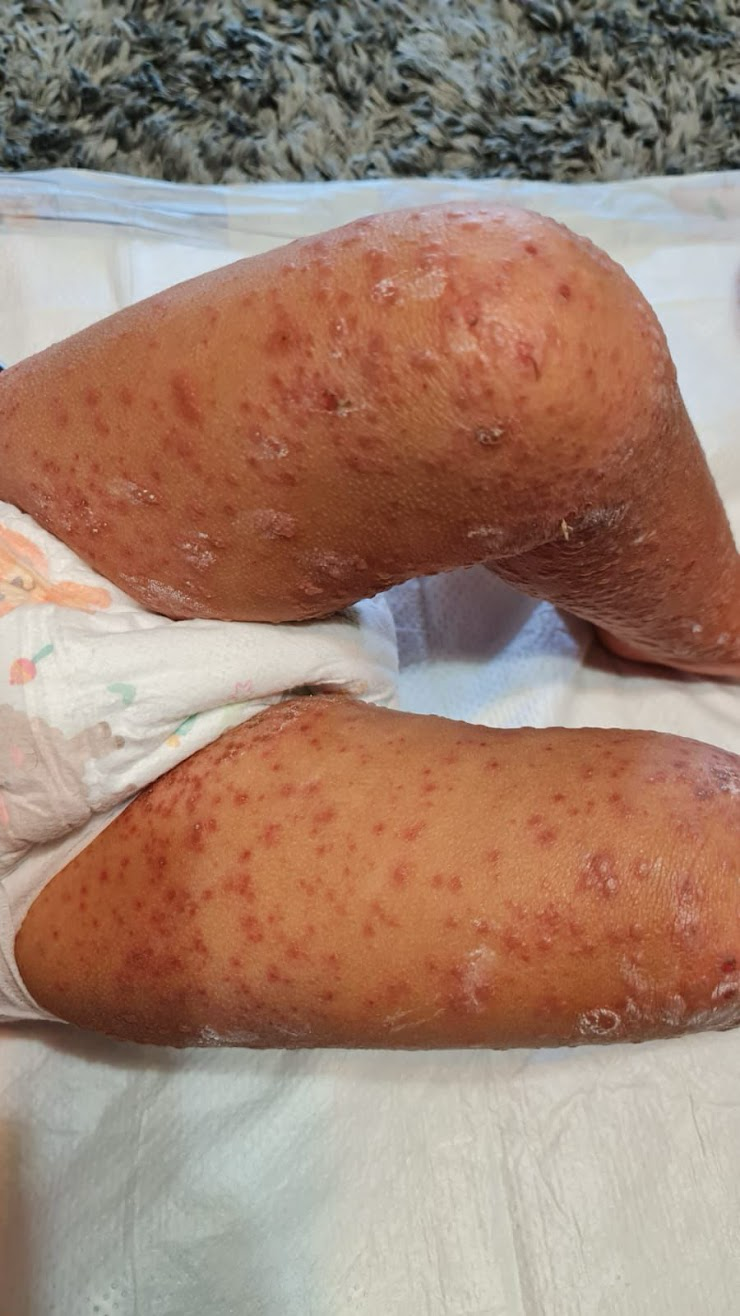


 **Enteroviral Disease**

 **Varicella (Chickenpox)**

 **Pityriasis Lichenoides et Varioliformis Acuta (PLEVA)**

 **Scabies**

 **Mpox (Monkeypox)**

1. A **14-month-old male patient** presented with a **maculopapular rash on different parts of the body since yesterday**, which **blanches on pressure**. He had a **history of gastroenteritis one week ago**. **What is your diagnosis?**


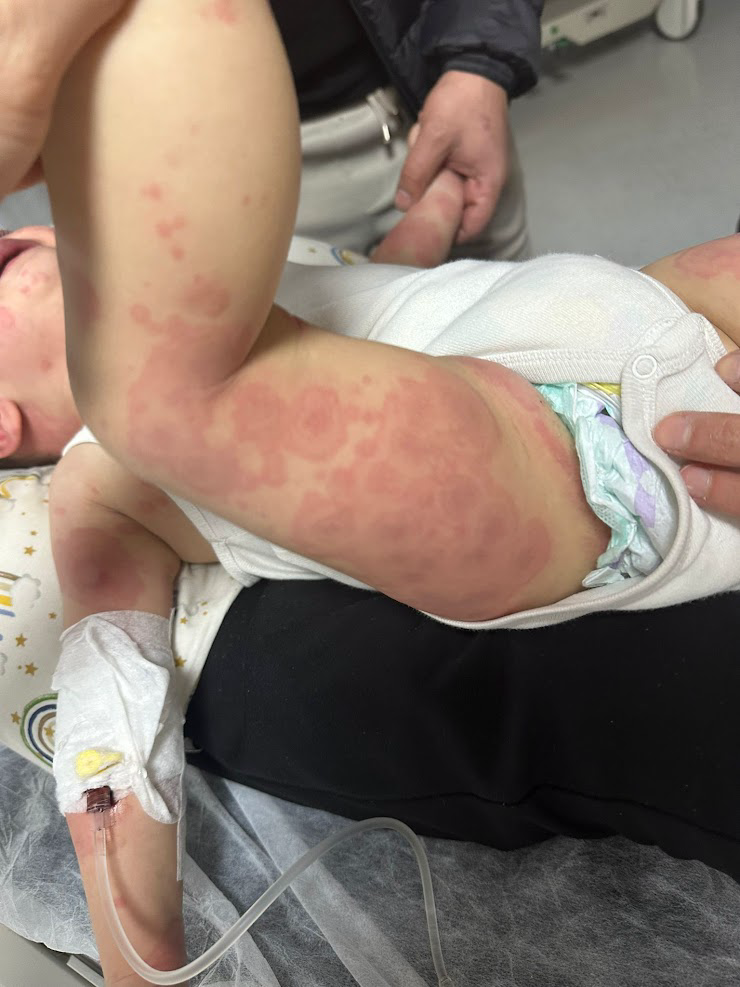


 **Urticaria**

 **Exanthema Subitum (Sixth Disease / Roseola)**

 **Parvovirus B19 Infection**

 **Erythema Multiforme**

 **Erythema Marginatum**

1. A **5-year-old male patient** presented with **painful red lesions on the extensor surfaces following a fever that had lasted for 3 days**. **What is your diagnosis?**


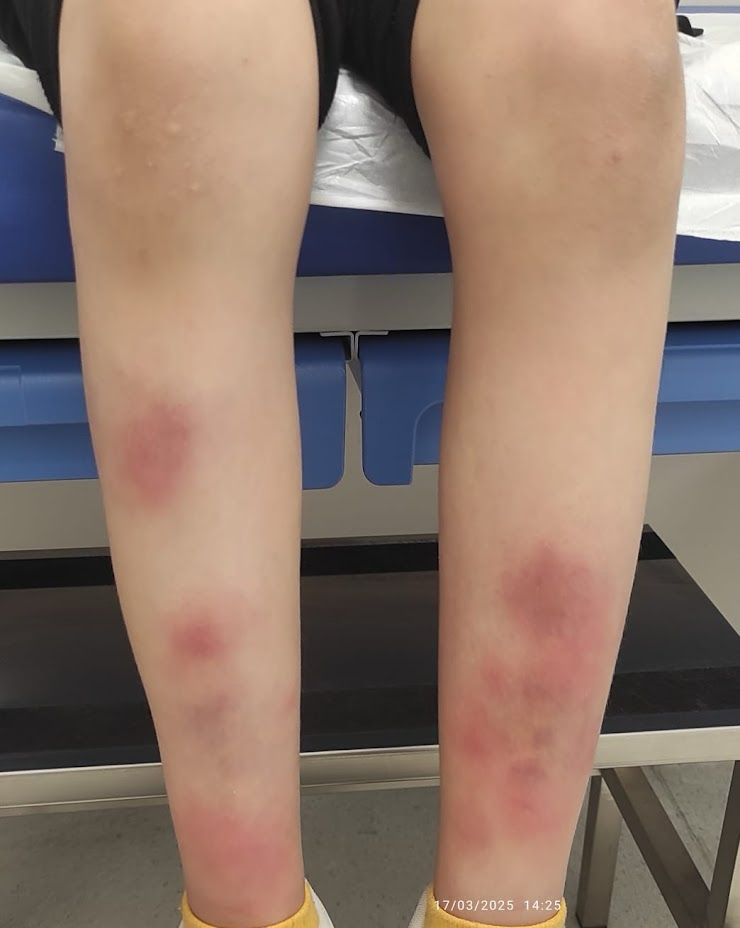


 **Subcutaneous Nodule**

 **Erythema Nodosum**

 **Henoch–Schönlein Purpura (HSP)**

 **Erythema Induratum**

 **Sarcoidosis**

1. A **14-month-old female patient** presented with a **rash that started today and has been progressively increasing**. She had **high fever for the past 3 days, which resolved today**. She has had **cough and runny nose for the past 2 days**. The **rash is widespread over the entire body**. **No additional findings were noted.** **What is your diagnosis?**


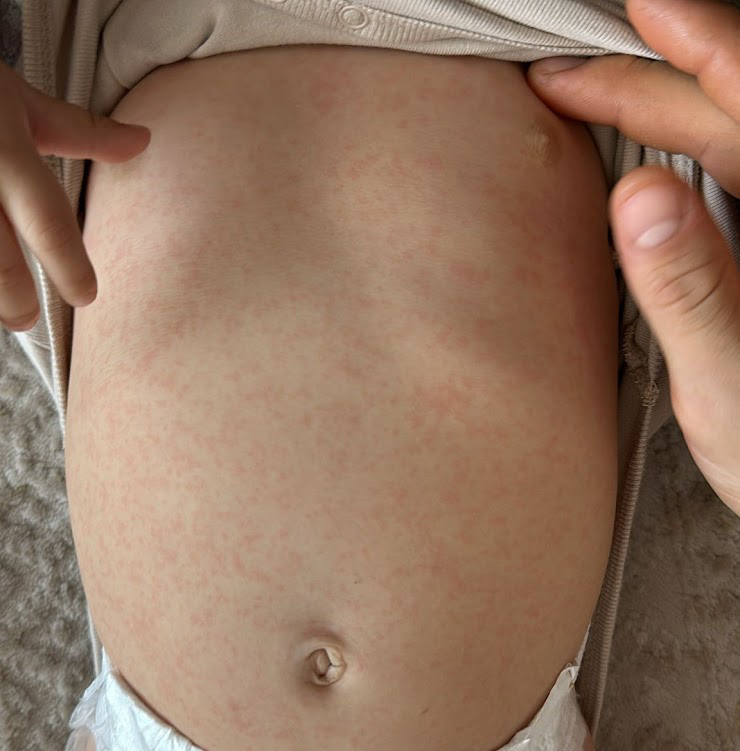


 **Measles**

 **Parvovirus B19 Infection (Fifth Disease)**

 **Enteroviral Exanthem**

 **Urticaria**

 **Exanthema Subitum (Sixth Disease / Roseola)**

1. A **5-year-old male patient** presented with **joint pain, swelling, edema on the dorsum of the feet, and a rash**. The **rash was predominantly distributed on the lower extremities**. **There was no fever.** **What is your diagnosis?**


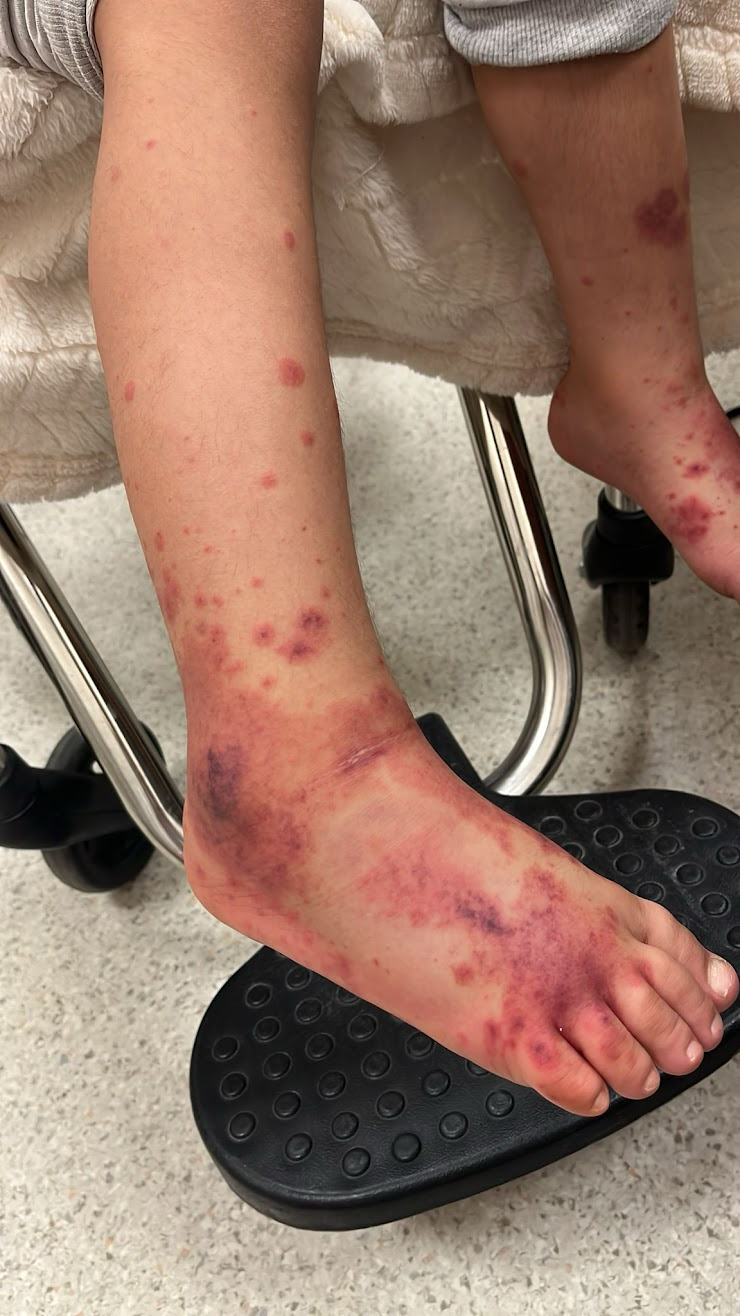


 **Subcutaneous Nodule**

 **Erythema Nodosum**

 **Henoch–Schönlein Purpura (HSP)**

 **Erythema Induratum**

 **Sarcoidosi**

1. A **5-year-old female patient** had been **taking trimethoprim–sulfamethoxazole for 6 days due to a urinary tract infection**. Her **rash started yesterday and spread over the entire body**. Her **temperature was 38.6°C**. **Blood pressure was normal**, and there was **no mucosal involvement**. **What is your diagnosis?**


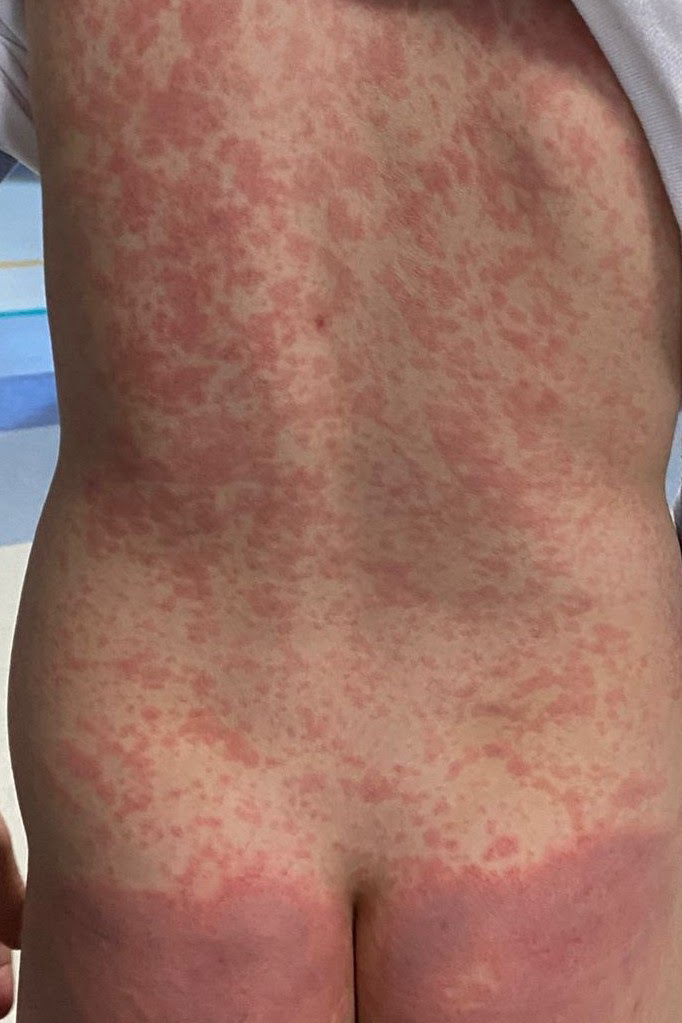


 **Henoch–Schönlein Purpura (HSP)**

 **Scarlet Fever**

 **Stevens–Johnson Syndrome**

 **Drug-Related Rash (Drug Eruption)**

 **Streptococcal Toxic Shock Syndrome**

1. A **7-year-old male patient** presented with **widespread hyperemic, itchy rashes around the mouth and nose on the face for approximately 2 days**. **This morning he had a fever of 37.5°C.** **What is your diagnosis?**


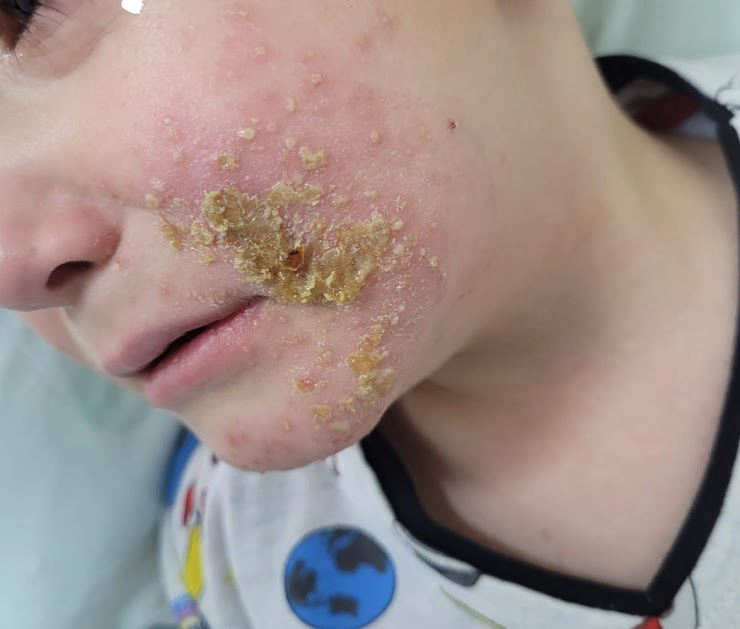


 **Human Papillomavirus (HPV) Infection**

 **Psoriasis**

 **Impetigo**

 **Seborrheic Dermatitis**

 **Tinea Faciei**

1. An **8-year-old male patient** presented with **fever, sore throat, and rash for the past 4 days**. **Strawberry tongue** was present. The **skin had a “gooseflesh” (sandpaper-like) appearance**. **What is your diagnosis?**


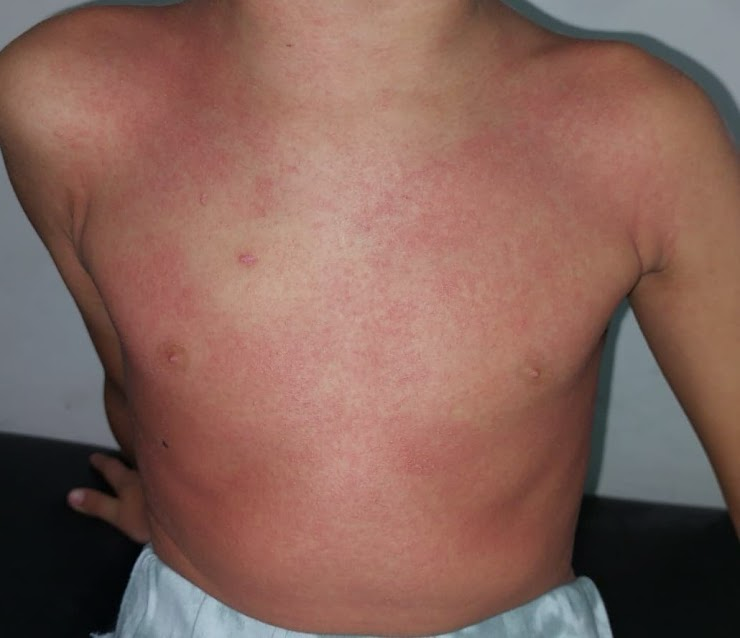


 **Henoch–Schönlein Purpura (HSP)**

 **Scarlet Fever**

 **Stevens–Johnson Syndrome**

 **Drug-Related Rash (Drug Eruption)**

 **Streptococcal Toxic Shock Syndrome**

1. A **17-year-old female patient** presented with **fever and rash that started 4 days ago**. Her **fever has been persistent**. **Conjunctivitis** is present and she also has a **cough**. The **rash is widespread over the entire body**. She is **unvaccinated**. **What is your diagnosis?**


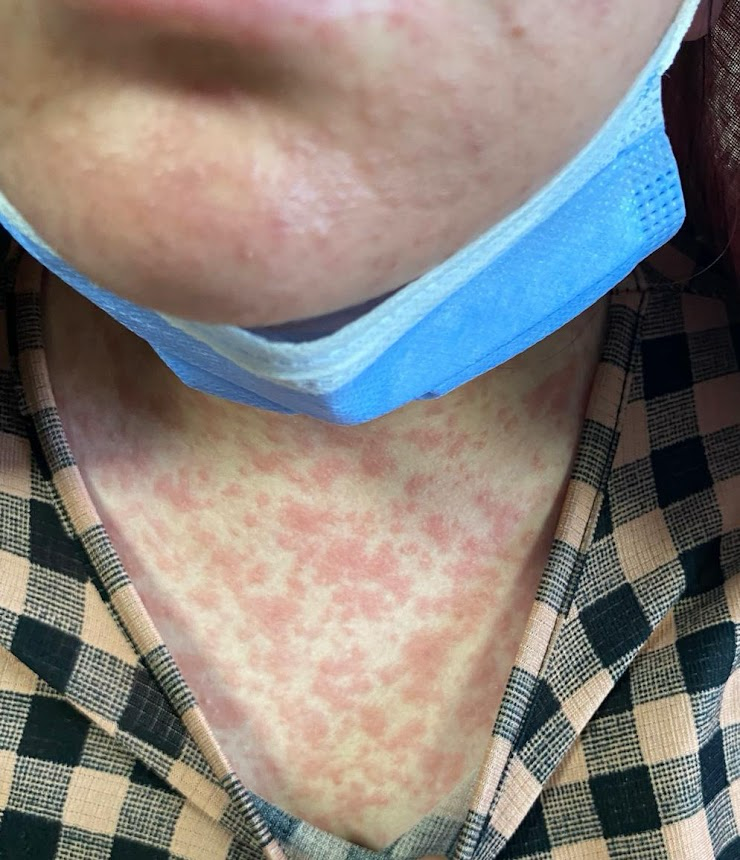


 **Parvovirus B19 Infection**

 **Measles**

 **Scarlet Fever**

 **Kawasaki Disease**

 **Sixth Disease (Exanthema Subitum / Roseola)**

1. A **6-year-old male patient** presented with a **painful skin lesion that had not improved despite 3 weeks of antibiotic treatment**. He **lives in the southeastern region**. When the **crust was removed, the center appeared necrotic**. **No additional complaints were reported.** **What is your diagnosis?**


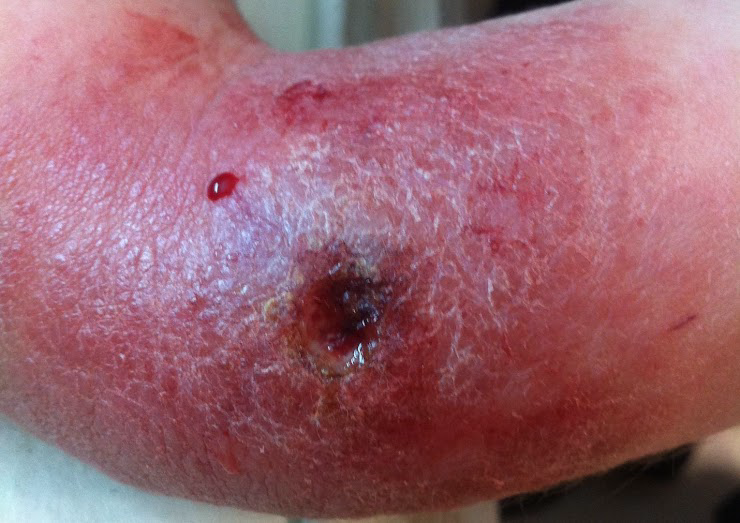


- **Ecthyma Gangrenosum**
- **Leishmaniasis**
- **Ulcerative Cellulitis**
- **Basal Cell Carcinoma**
- **Granuloma Annulare**

1. A **22-month-old female patient** presented with **fever and a widespread rash over the entire body since yesterday**. The **patient was hypotensive and appeared lethargic (drowsy)**. Her **temperature was 39°C**. **What is your diagnosis?**


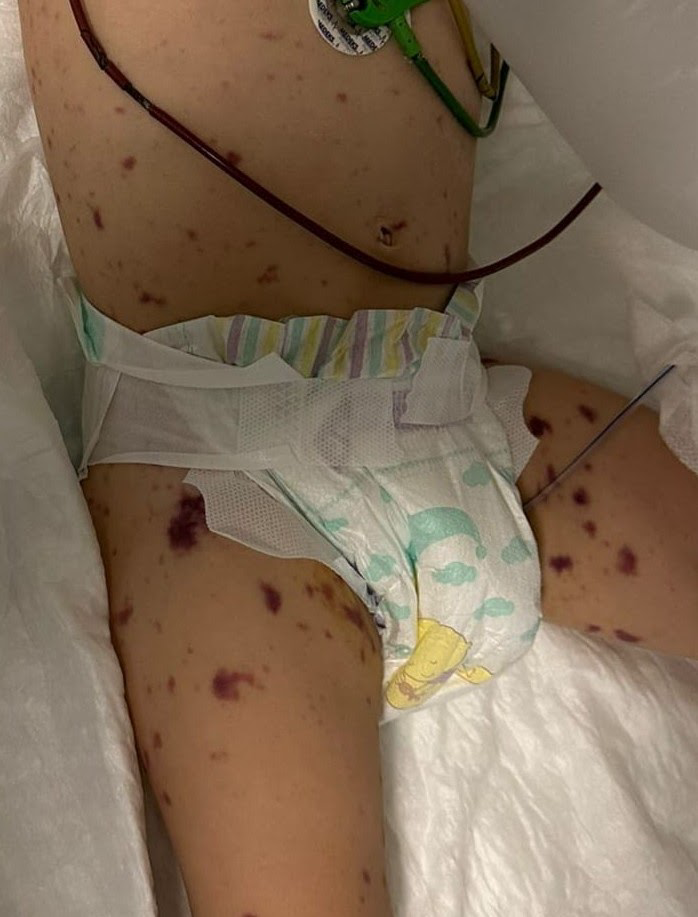


 **Meningococcemia**

 **Henoch–Schönlein Purpura (HSP)**

 **Acute Infantile Hemorrhagic Edema**

 **Cryoglobulinemia**

 **Rickettsiosis**

1. A **16-year-old male patient** reported that they had been **repairing their adobe house in the village**, and **on the same day the skin lesions shown in the image began to appear**. There were **no signs of meningeal irritation**, and **blood pressure was normal**. In addition, **a few of the lesions were vesicle-like**. **Pruritus (itching) was present**, and the **rash was widespread over the entire body**. **What is your diagnosis?**


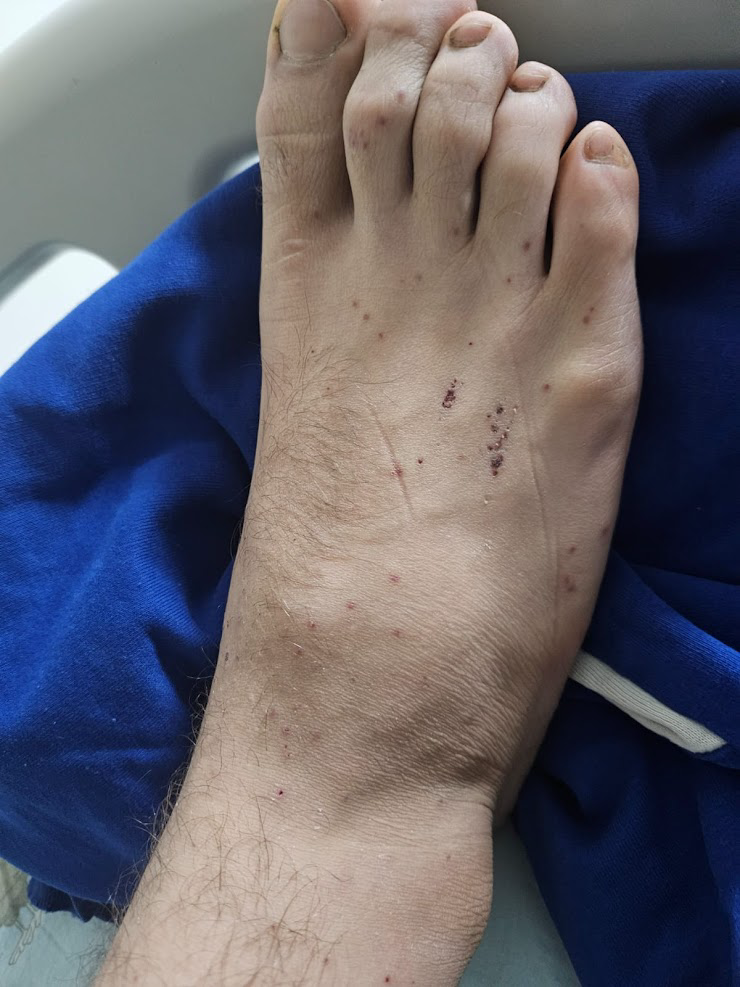


 **Insect Bite**

 **Meningococcemia**

 **Henoch–Schönlein Purpura (HSP)**

 **Immune Thrombocytopenia (ITP)**

 **Acute Infantile Hemorrhagic Edema**

1. A **12-year-old male patient** presented with **sore throat, fever, and rash for the past 2 days**. The **rash was widespread over the entire body**. **Redness was present in the oropharynx**. **What is your diagnosis?**


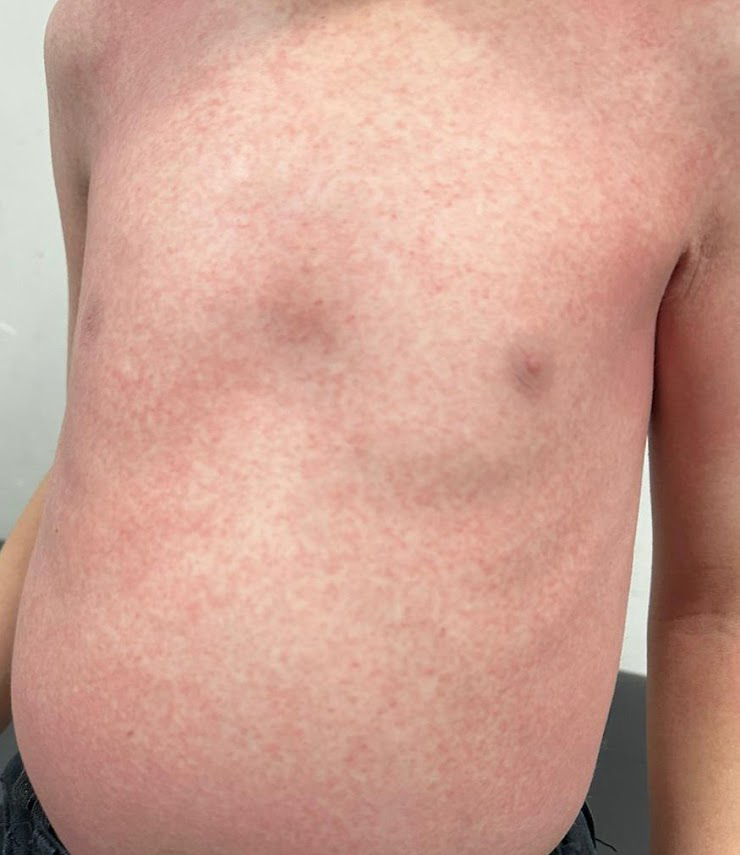


 **Streptococcal Toxic Shock Syndrome**

 **Measles**

 **Scarlet Fever**

 **Parvovirus B19 Infection**

 **Infectious Mononucleosis**

1. A **4-year-old female patient** presented with a **rash that has been appearing and disappearing on different parts of the body for the past 2 days**. Her **eyes were edematous**. The **lesions were plaque-like**. **No additional complaints were reported.** **What is your diagnosis?**


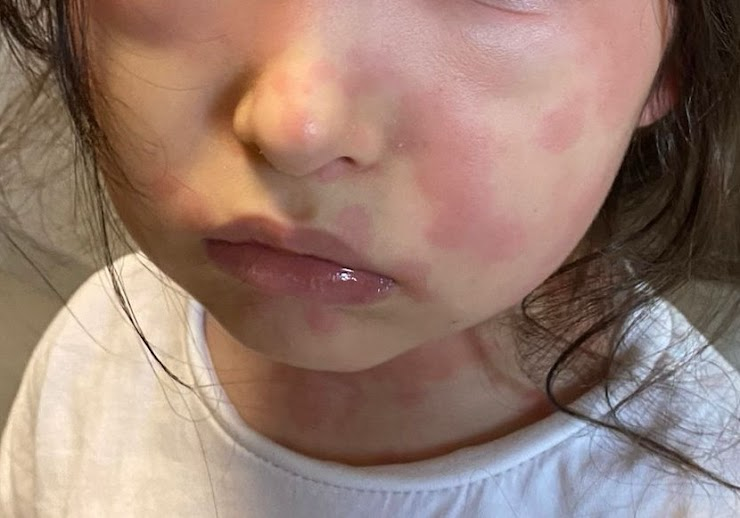


 **Parvovirus B19 Infection**

 **Urticaria**

 **Measles**

 **Urticarial Vasculitis**

 **Rubella**

1. A **2-year-old female patient** presented with **widespread skin lesions on the trunk that have been present for 2 months and do not tend to coalesce**. **No additional complaints were reported.** **What is your diagnosis?**


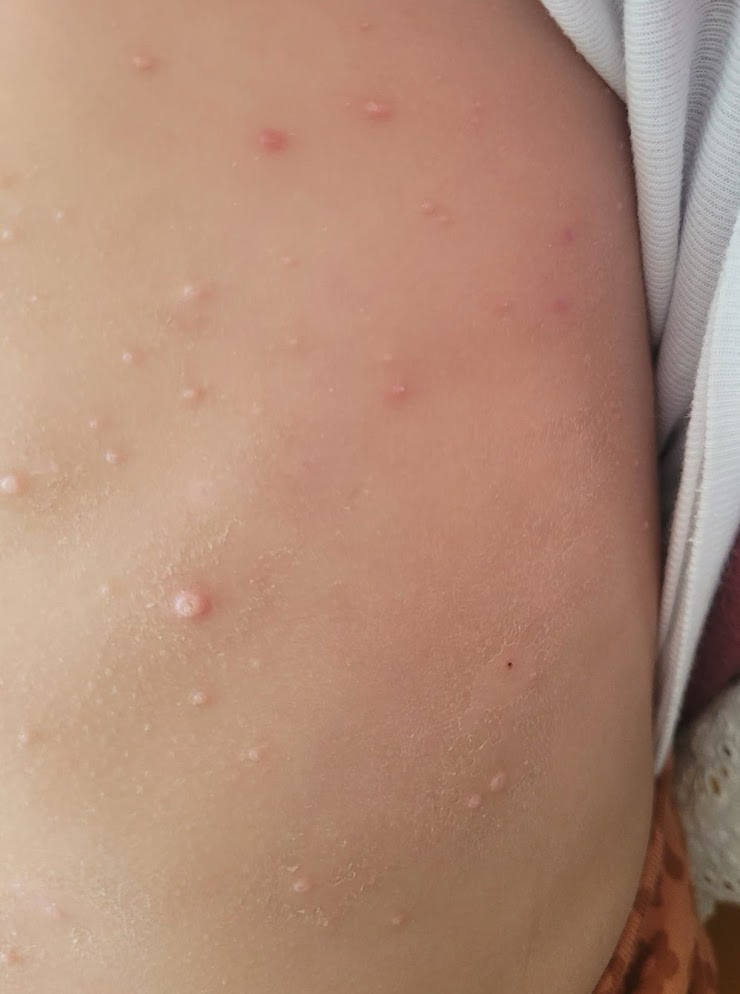


 **Milia**

 **Verruca Vulgaris (Common Warts)**

 **Varicella (Chickenpox)**

 **Molluscum Contagiosum**

 **Pityriasis Lichenoides et Varioliformis Acuta (PLEVA)**

1. A **13-year-old male patient** presented with **fever, runny nose, and rash since yesterday**. He appeared **fatigued**, and his **blood pressure was near the lower limit of normal**. His **consciousness was clear**. **What is your diagnosis?**

**
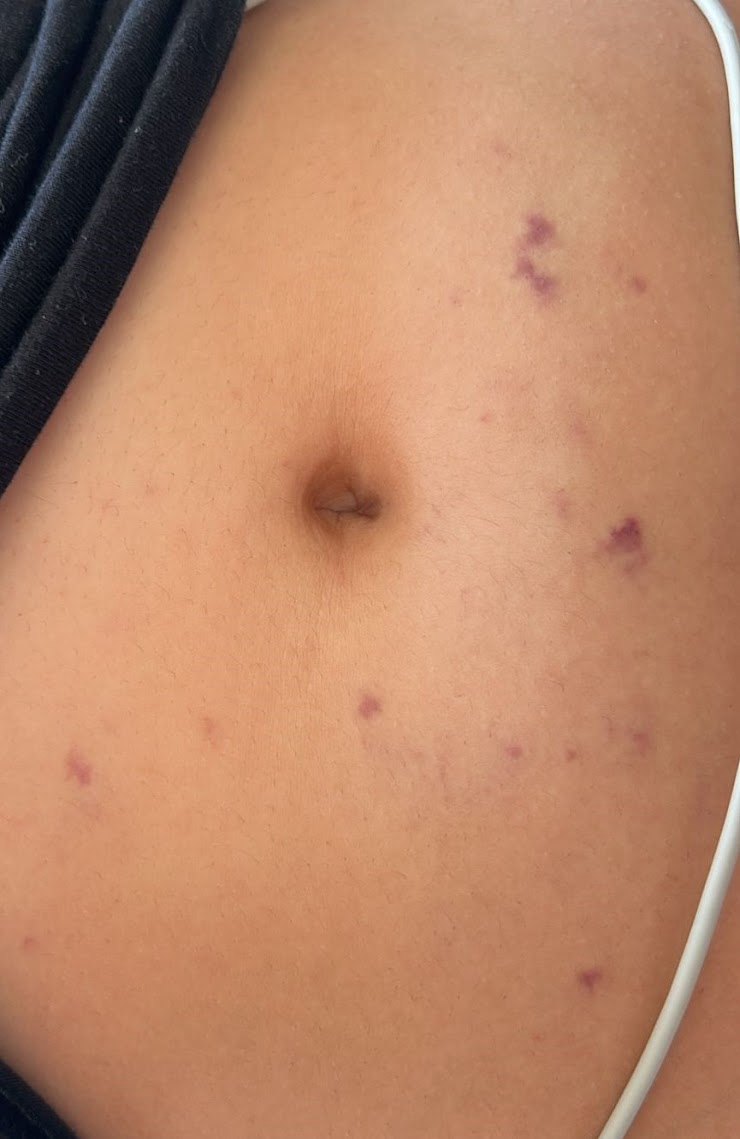
**

 **Meningococcemia**

 **Trauma-related rash**

 **Immune Thrombocytopenia (ITP)**

 **Enteroviral Rash (Enteroviral Exanthem)**

 **Streptococcal Toxic Shock Syndrome**

1. A **4-year-old female patient** had been **using an antiepileptic medication for 2 months**. She presented with **a progressively worsening widespread rash over the body for the past 3–4 days**, along with **dryness and cracking of the lips**. **Conjunctivitis** was present in the eyes. She had **fever for the past 2–3 days**. **Areas of skin desquamation (peeling)** were also observed. **What is your diagnosis?**

**
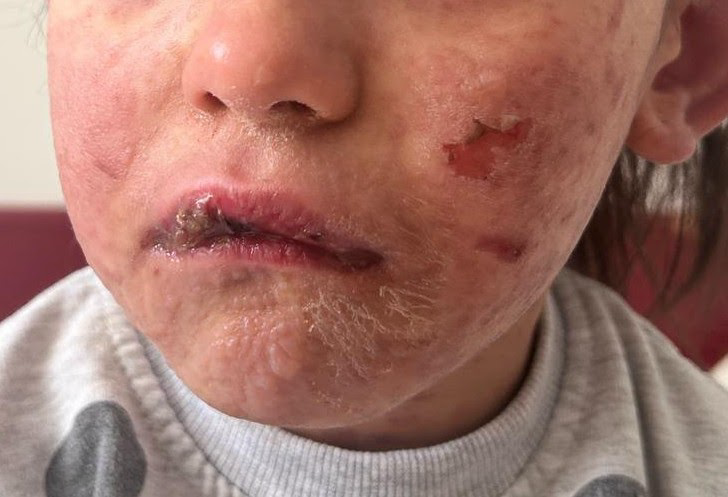
**

 **Drug Rash with Eosinophilia and Systemic Symptoms (DRESS)**

 **Acute Generalized Exanthematous Pustulosis (AGEP)**

 **Stevens–Johnson Syndrome**

 **Streptococcal Toxic Shock Syndrome**

 **Purpura Fulminans**

1. A **1-month-old male patient** presented with a **rash that has been present for 9 days**. **There was no fever** and **no additional complaints**. **What is your diagnosis?**


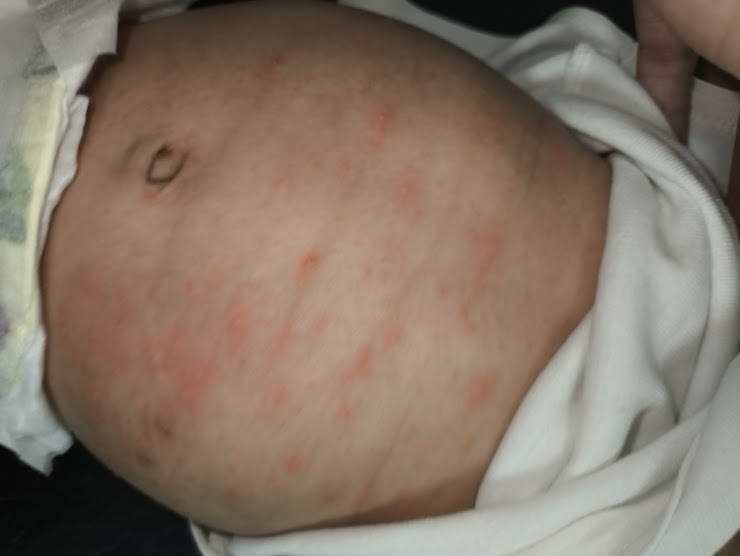


 **Erythema Toxicum (Toxic Erythema of the Newborn)**

 **Atopic Dermatitis**

 **Scabies**

 **Varicella (Chickenpox)**

 **Milia**

1. A **5-year-old male patient** presented with a **rash that has been appearing and disappearing on different parts of the body for the past 3 days**, with **similar characteristics each time**. **What is your diagnosis?**


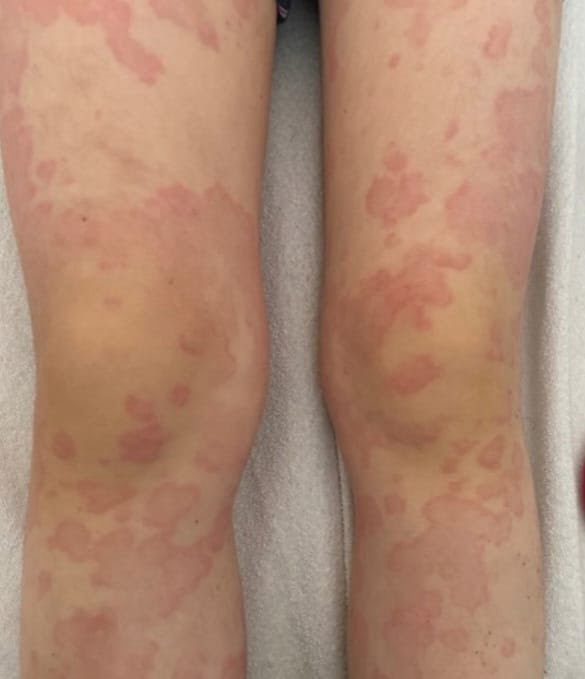


 **Urticaria**

 **Varicella (Chickenpox)**

 **Measles**

 **Urticarial Vasculitis**

 **Erythema Multiforme**

1. A **7-year-old male patient** presented with **fever and a widespread rash over the entire body for the past 2 days**, with a **tendency for the lesions to coalesce**. The **flexural areas were pale**. He **appeared fatigued**. **Blood pressure was normal**, and his **temperature was 38.6°C**. **What is your diagnosis?**


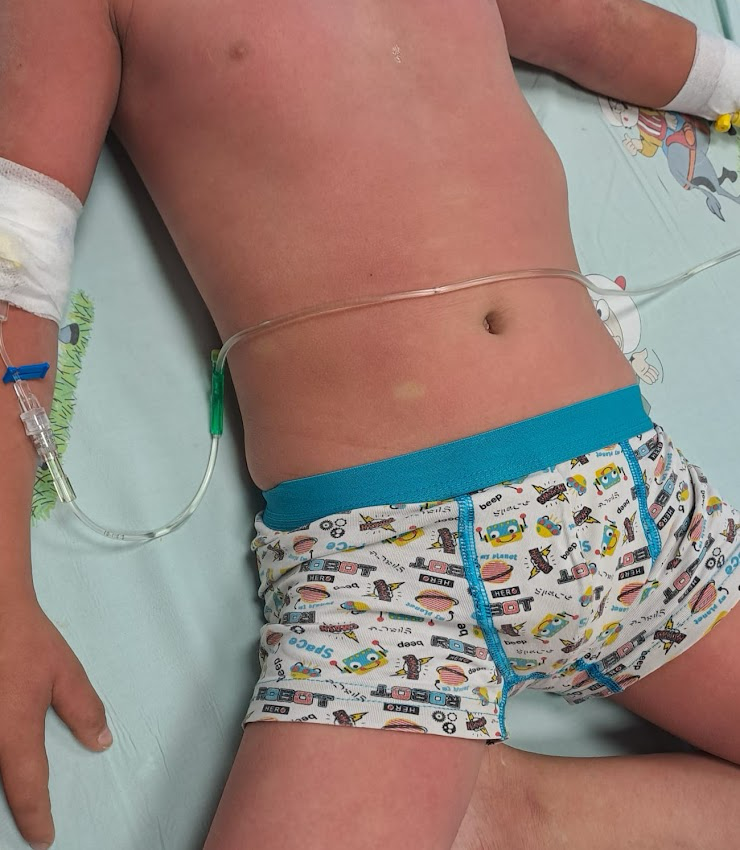


 **Scarlet Fever**

 **Measles**

 **Parvovirus B19 Infection**

 **Streptococcal Toxic Shock Syndrome**

 **Kawasaki Disease**

1. A **14-year-old male patient** presented with a **widespread rash over the entire body for the past 3 days**. The **rash was also present on the scalp**. He had **fever and fatigue** as well. **What is your diagnosis?**


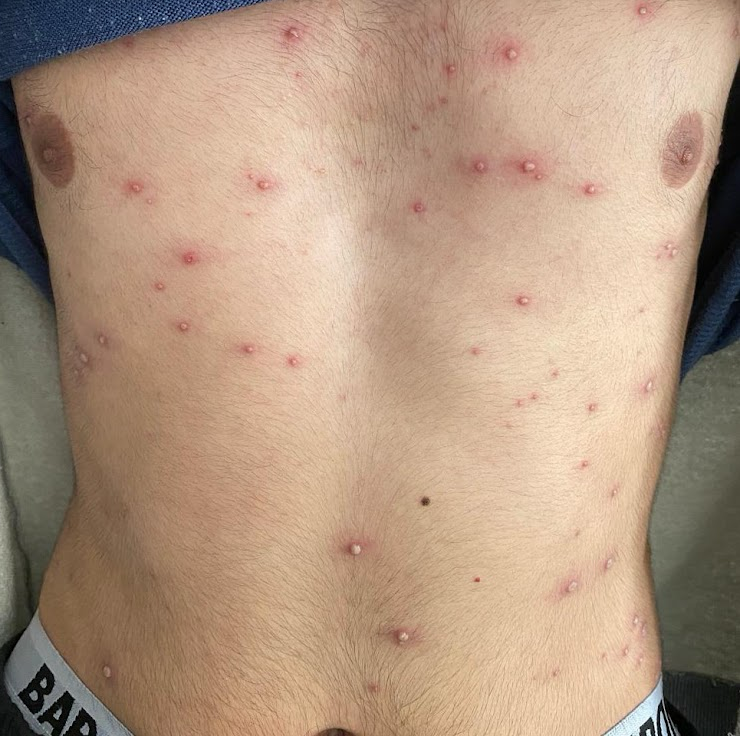


 **Pityriasis Lichenoides et Varioliformis Acuta (PLEVA)**

 **Insect Bite**

 **Varicella (Chickenpox)**

 **Molluscum Contagiosum**

 **Herpes Simplex Virus Infection**

1. A **2-year-old female patient** presented with a **lesion in the gluteal region that has been present for 1 month**. **No additional findings were noted.** **What is your diagnosis?**


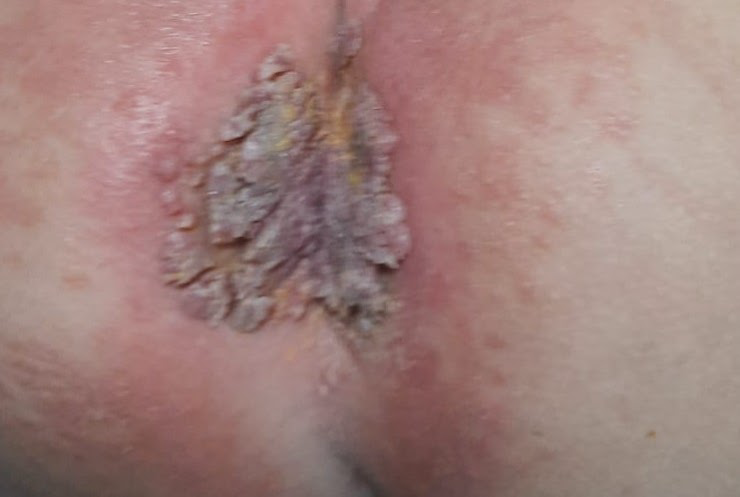


 **Condyloma (Genital Warts)**

 **Molluscum Contagiosum**

 **Sebaceous Hyperplasia**

 **Basal Cell Carcinoma**

 **Syringoma**

1. A **9-year-old male patient** presented with **sore throat, fever, and rash for the past 5 days**. His **blood pressure was within the normal range for his age**.**What is your diagnosis?**


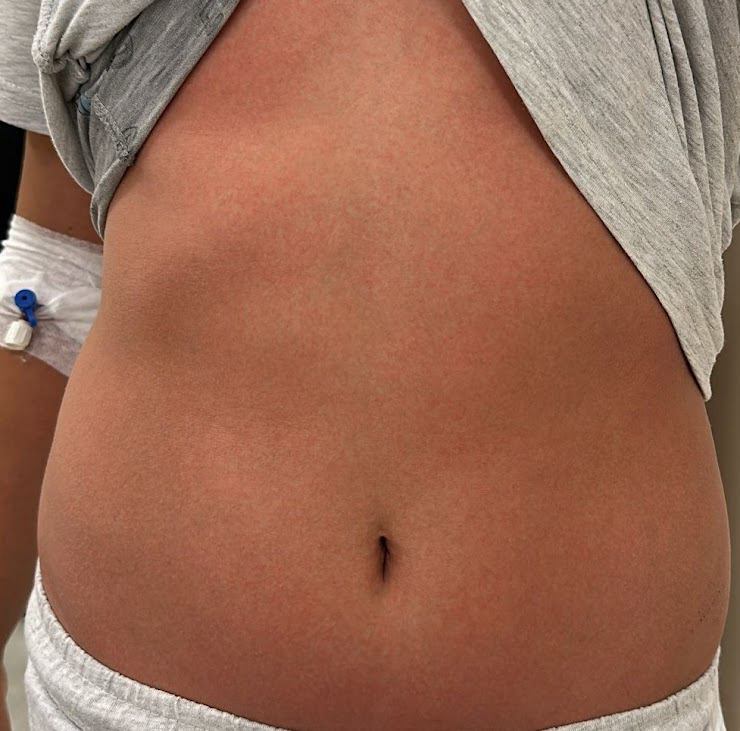


 **Scarlet Fever**

 **Parvovirus B19 Infection**

 **Staphylococcal Toxic Shock Syndrome**

 **Infectious Mononucleosis**

 **Kawasaki Disease**

1. A **14-year-old male patient** presented with a **rash on the left side of the abdomen that extends toward the back**. He **has pain**. **No additional complaints were reported.** **What is your diagnosis?**


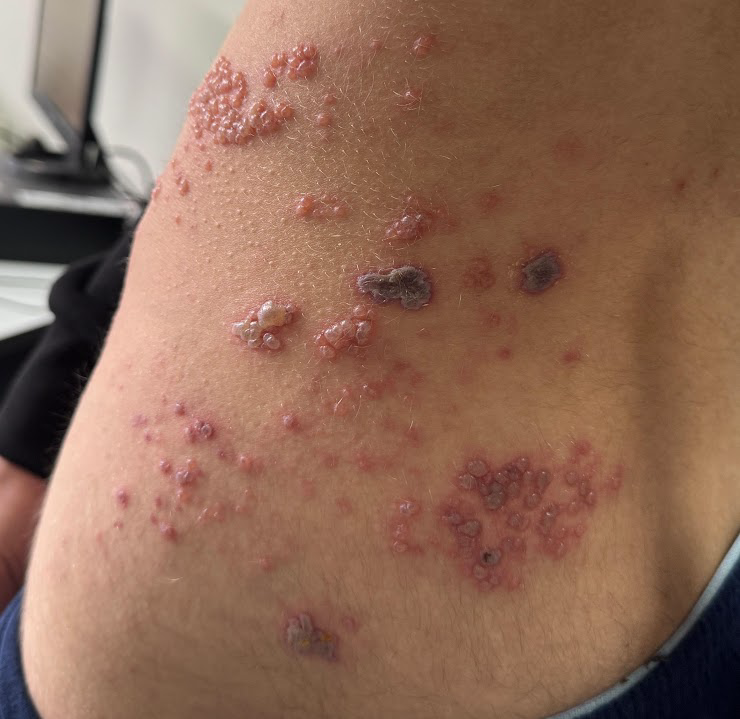


 **Varicella (Chickenpox)**

 **Herpes Simplex Virus Infection**

 **Dermatitis Herpetiformis**

 **Herpes Zoster (Shingles)**

 **Bullous Impetigo**

1. A **16-month-old male patient** presented with **widespread redness on the body for the past 3 days and some dark-colored skin lesions**. **Most of the lesions did not blanch on pressure**. The **dorsum of the hands was edematous**. **No additional findings were noted.** **What is your diagnosis?**


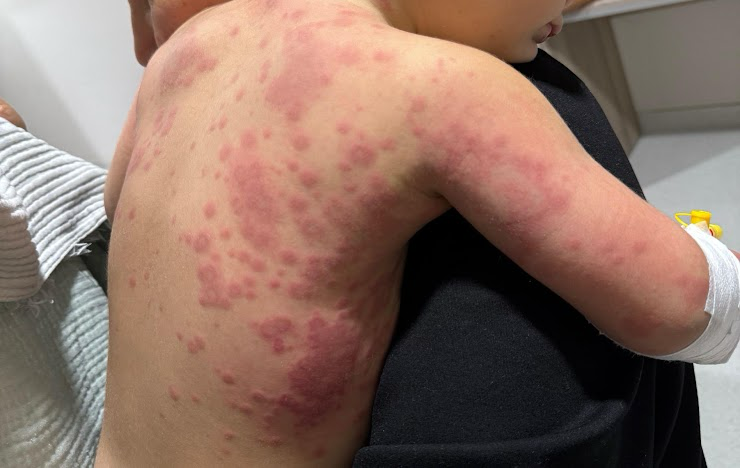


 **Kawasaki Disease**

 **Henoch–Schönlein Purpura (HSP)**

 **Drug Rash with Eosinophilia and Systemic Symptoms (DRESS)**

 **Acute Infantile Hemorrhagic Edema**

 **Meningococcemia**

1. An **8-year-old male patient** presented with **fever and rash for the past 2 days**. His **fever was persistent**. The **rash was present on the trunk, arms, and legs**. He had been **taking cefixime for the past 4 days**. **Other system examinations were normal.** **What is your diagnosis?**


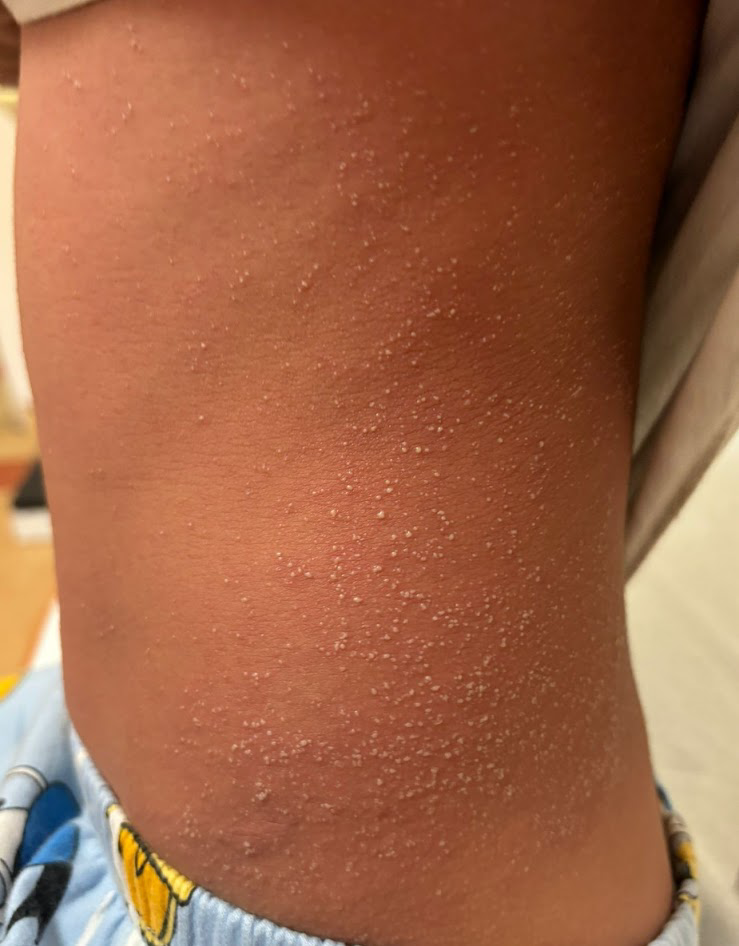


 **Gianotti–Crosti Syndrome**

 **Scarlet Fever**

 **Psoriasis**

 **Acute Generalized Exanthematous Pustulosis (AGEP)**

 **Drug Rash with Eosinophilia and Systemic Symptoms (DRESS)**

1. A **4-year-old female patient** presented with a **rash for the past 3 days**, with **similar lesions on the gluteal region, hands, and feet**. **Fever was present.** **What is your diagnosis?**


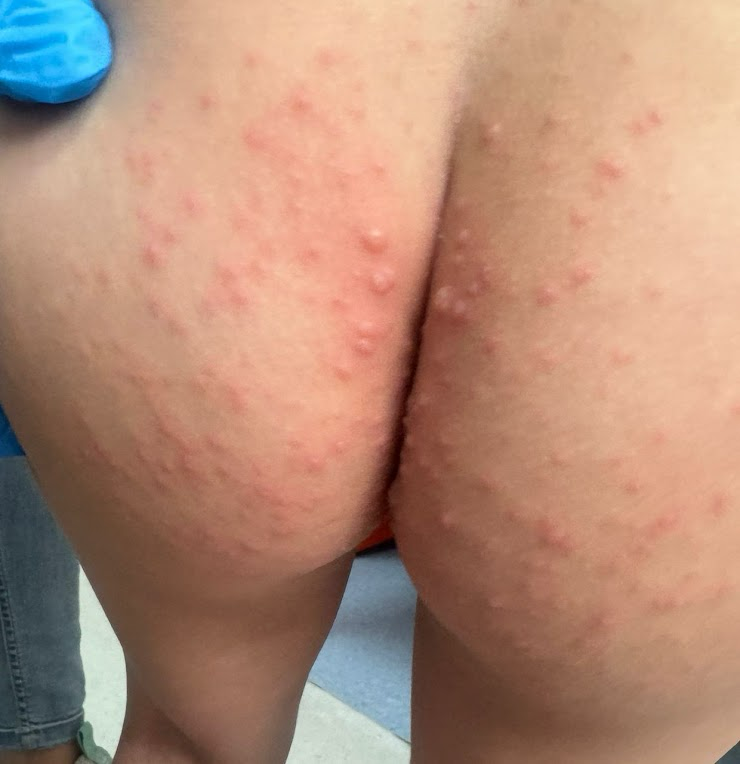


 **Varicella (Chickenpox)**

 **Enteroviral Rash (Enteroviral Exanthem)**

 **Herpes Zoster (Shingles)**

 **Molluscum Contagiosum**

 **Herpes Simplex Virus Infection**

1. A **6-year-old male patient** presented with **fever for 4 days and a rash for 2 days**. He had been **taking cefixime for the past 2 days**. The **patient had similar rashes on the hands and feet**. **What is your diagnosis?**


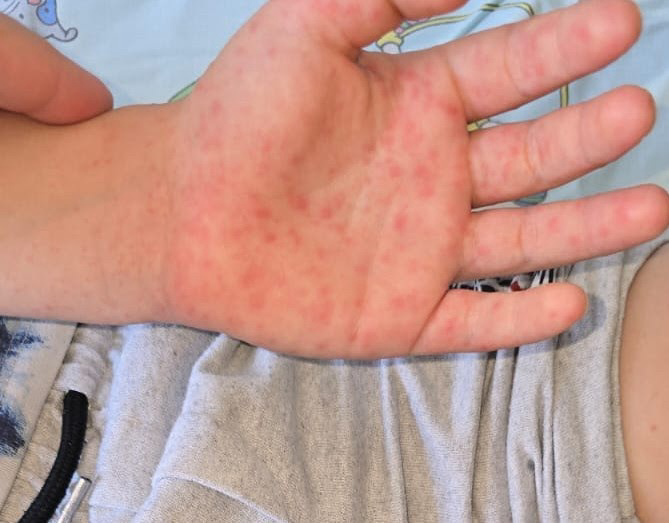


 **Drug-related rash (drug eruption)**

 **Varicella (Chickenpox)**

 **Herpes Zoster (Shingles)**

 **Enteroviral Rash (Enteroviral Exanthem)**

 **Parvovirus B19 Infection**

1. An **8-year-old male patient** presented with **joint pain, abdominal pain, and rash**. **What is your diagnosis?**


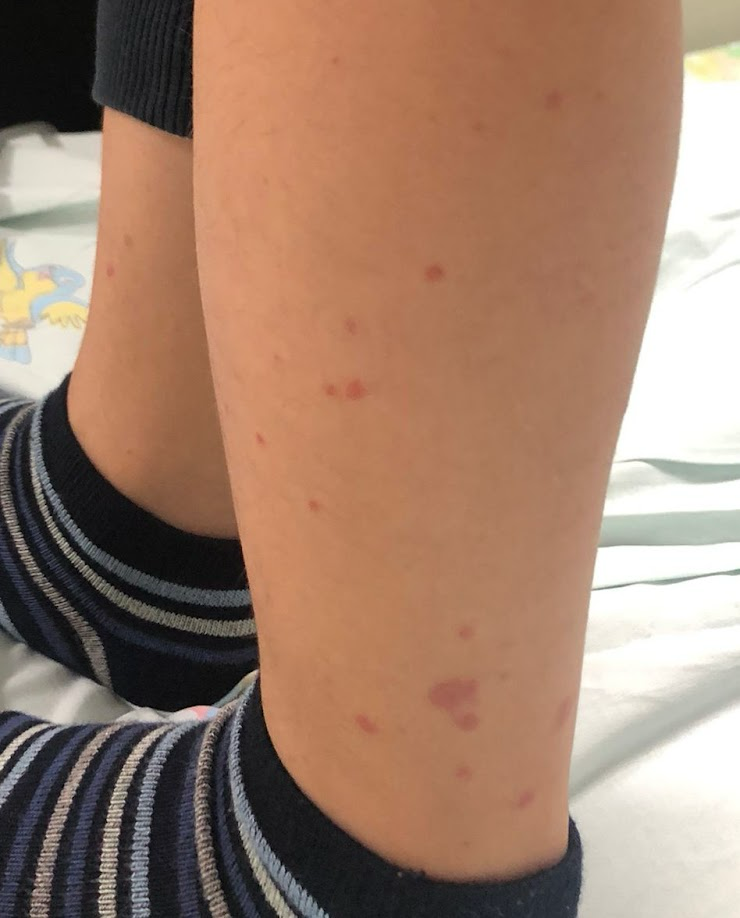


 **Henoch–Schönlein Purpura (HSP)**

 **Acute Infantile Hemorrhagic Edema (AIHE)**

 **Meningococcemia**

 **Familial Mediterranean Fever**

 **Kawasaki Disease**

1. A **4-year-old female patient** had been **taking carbamazepine for 15 days due to epilepsy**. She presented with a **rash that started yesterday and spread over the entire body**. Her **temperature was 38.6°C**. **There was no mucosal involvement.** **What is your diagnosis?**


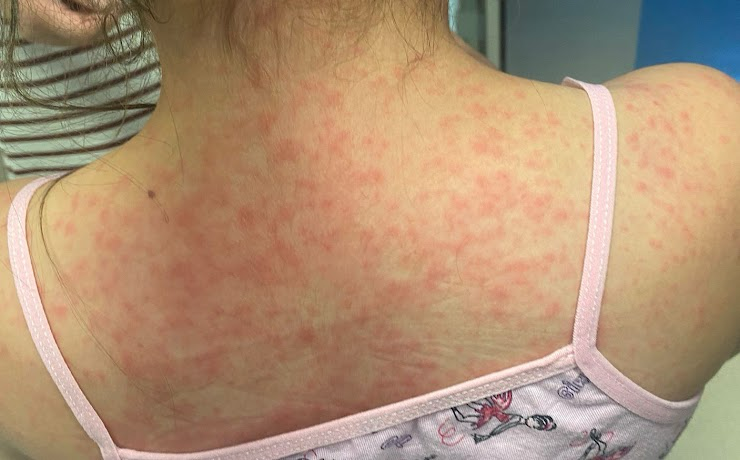


 **Stevens–Johnson Syndrome**

 **Drug-related rash (drug eruption)**

 **Pemphigus Vulgaris**

 **Kawasaki Disease**

 **ANCA-associated Vasculitis**

1. A **4-year-old male patient** presented with **fever and rashes that have been appearing and disappearing on different parts of the body for the past 4 days**. There was **no rash in the nasolabial sulcus**. There was **no known allergy**. **What is your diagnosis?**


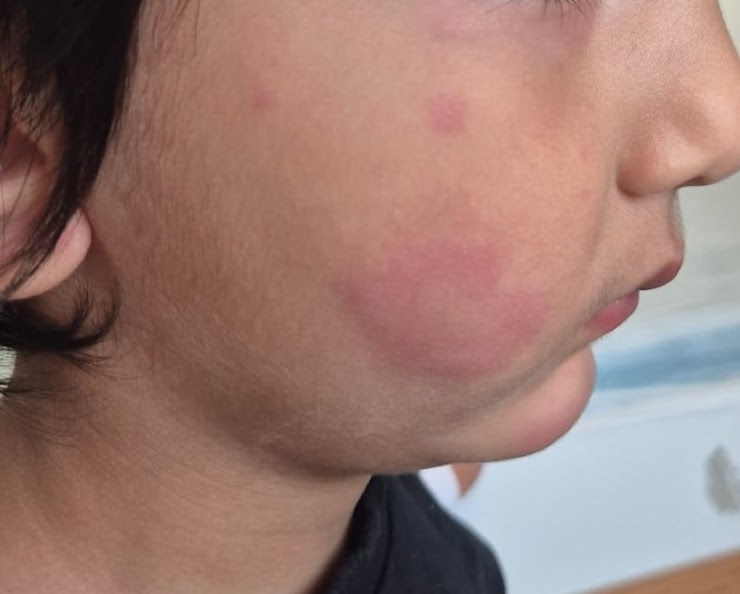


 **Urticaria**

 **Measles**

 **Parvovirus B19 Infection**

 **Erythema Multiforme**

 **Erythema Marginatum**

1. A **4-year-old male patient** presented with **itchy and painful lesions that have been present for 2 days and are distributed along a dermatome**. **What is your diagnosis?**


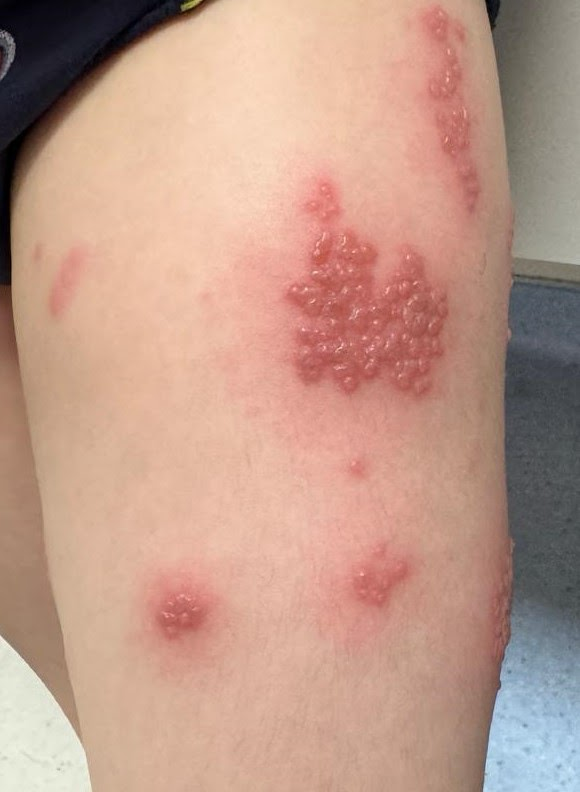


 **Herpes Zoster (Shingles)**

 **Varicella (Chickenpox)**

 **Contact Dermatitis**

 **Bullous Impetigo**

 **Scabies**

1. A **7-month-old male patient** presented with **fever, rash, runny nose, and cough for the past 2 days**. The **rash started on the head and neck and spread to the entire body**. There was **no edema of the hands or feet**, and **no lymphadenopathy** was detected. The patient **appeared fatigued**. **Conjunctivitis** was present. The **cough was productive**. The **fever had been persistent/resistant**. **What is your diagnosis?**


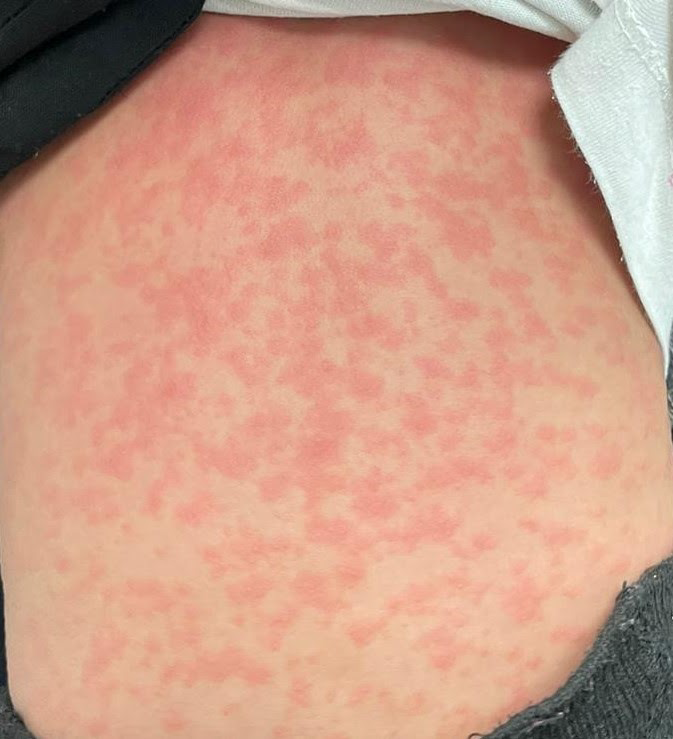


 **Scarlet Fever**

 **Kawasaki Disease**

 **Measles**

 **Exanthema Subitum (Sixth Disease / Roseola)**

 **Parvovirus B19 Infection**

1. A **12-year-old male patient** presented with **fever, sore throat, and rash for the past 2 days**. The **oropharynx was hyperemic**. **Pruritus (itching)** was present. The **skin had a rough (sandpaper-like) texture**. **What is your diagnosis?**


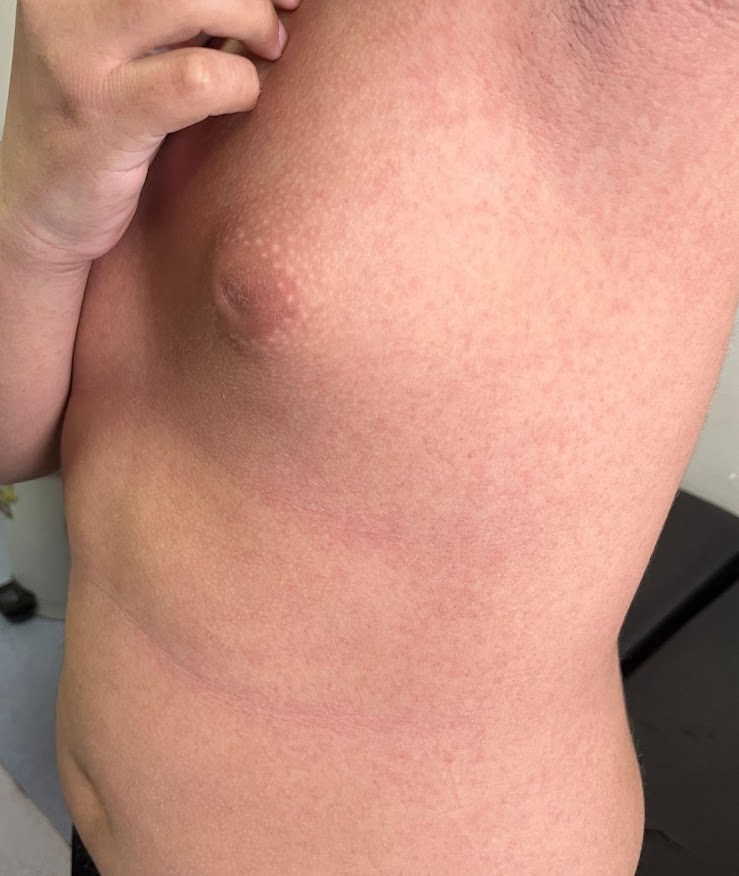


- **Parvovirus B19 Infection**
- **Scarlet Fever**
- **Kawasaki Disease**
- **Infectious Mononucleosis**
- **Acute Generalized Exanthematous Pustulosis (AGEP)**

1. A **16-year-old male patient** presented with **rash and fatigue for the past 3 days**. **What is your diagnosis?**


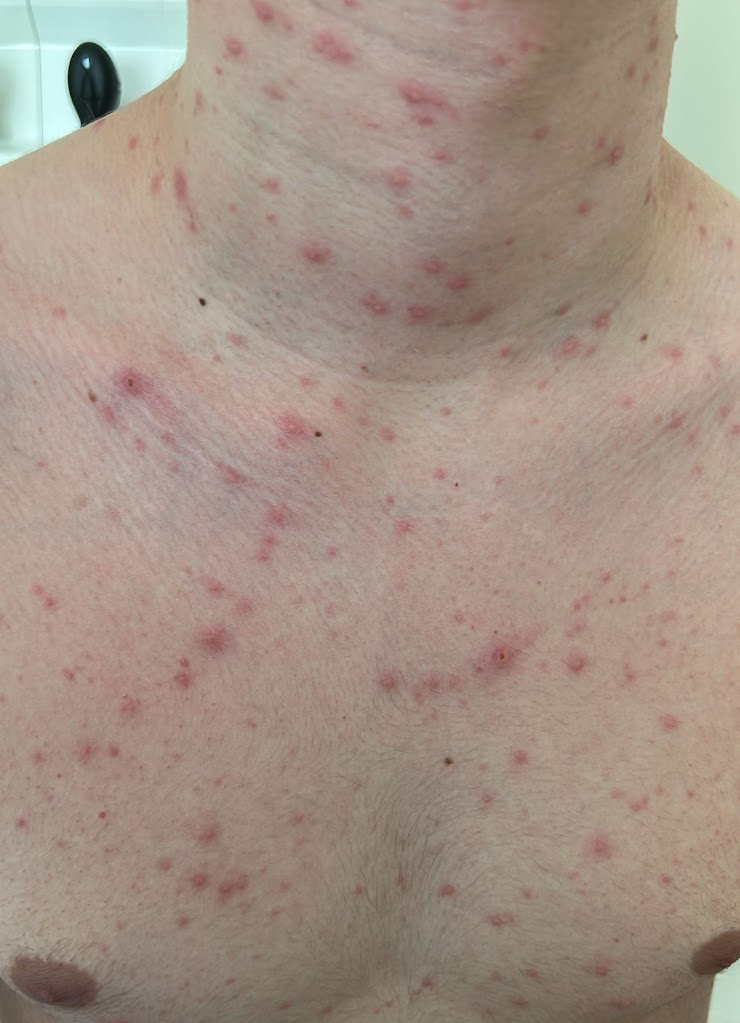


 **Acne**

 **Enteroviral Rash (Enteroviral Exanthem)**

 **Drug Rash with Eosinophilia and Systemic Symptoms (DRESS)**

 **Pityriasis Lichenoides et Varioliformis Acuta (PLEVA)**

 **Varicella (Chickenpox)**

1. A **4-year-old male patient** presented with a **rash on the face that has been present for the past 4 days**. **What is your diagnosis?**


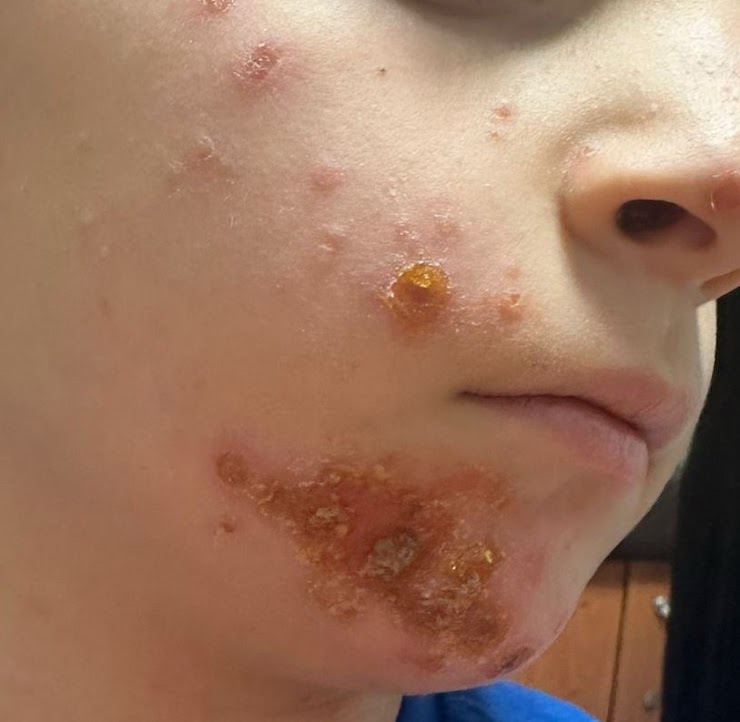


 **Herpes Zoster (Shingles)**

 **Cellulitis**

 **Impetigo**

 **Contact Dermatitis**

 **Atopic Dermatitis**

1. A **10-year-old male patient** presented with **rash spreading over the entire body for 1 day, fever, and decreased urine output**. His **blood pressure was low**, and **capillary refill time was 5–6 seconds**. **What is your diagnosis?**


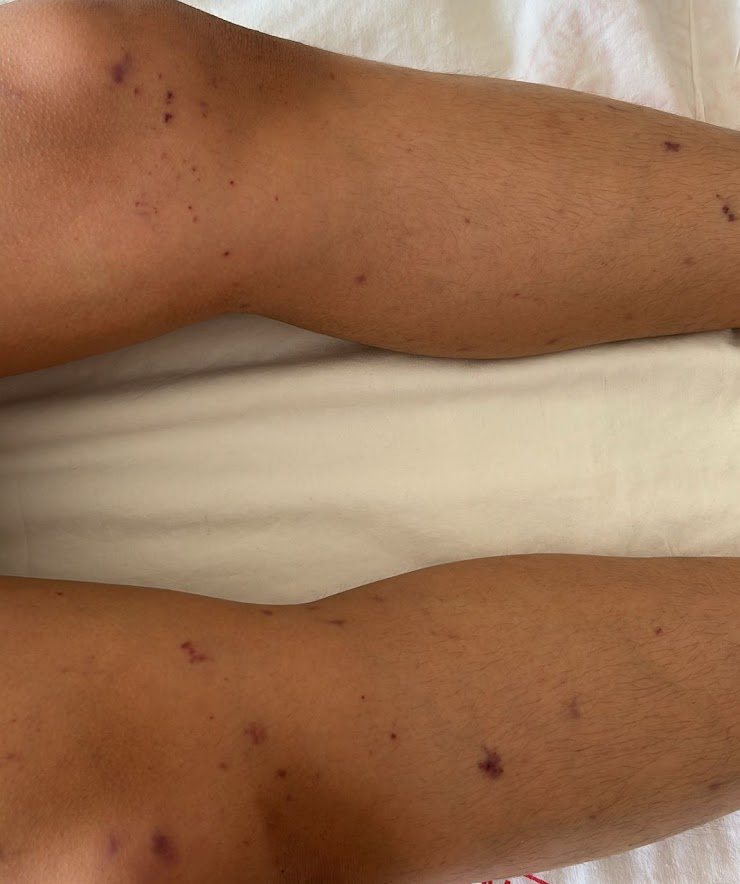


 **Henoch–Schönlein Purpura (HSP)**

 **Meningococcemia**

 **Urticarial Vasculitis**

 **Hemolytic Uremic Syndrome (HUS)**

 **Scarlet Fever**

41- A **3-month-old male patient** presented with **skin lesions that have been present for approximately 1 month**.**What is your diagnosis?**


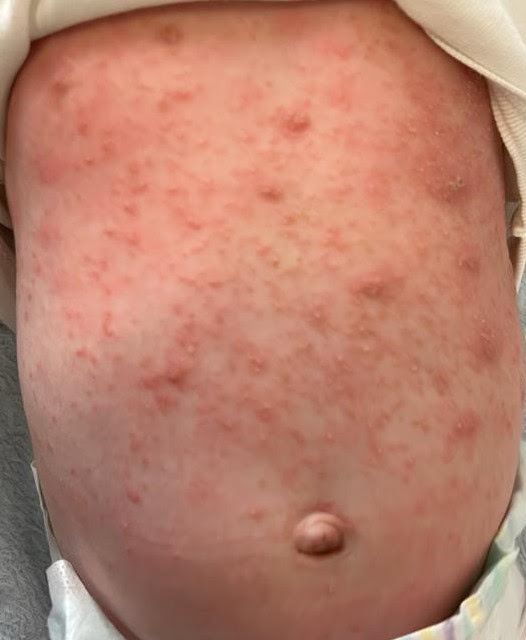


 **Atopic Dermatitis**

 **Pityriasis Lichenoides et Varioliformis Acuta (PLEVA)**

 **Varicella (Chickenpox)**

 **Scabies**

 **Enteroviral Rash (Enteroviral Exanthem)**

42- A **3-year-old male patient** presented with **rash and itching since yesterday**.

**What is your diagnosis?**


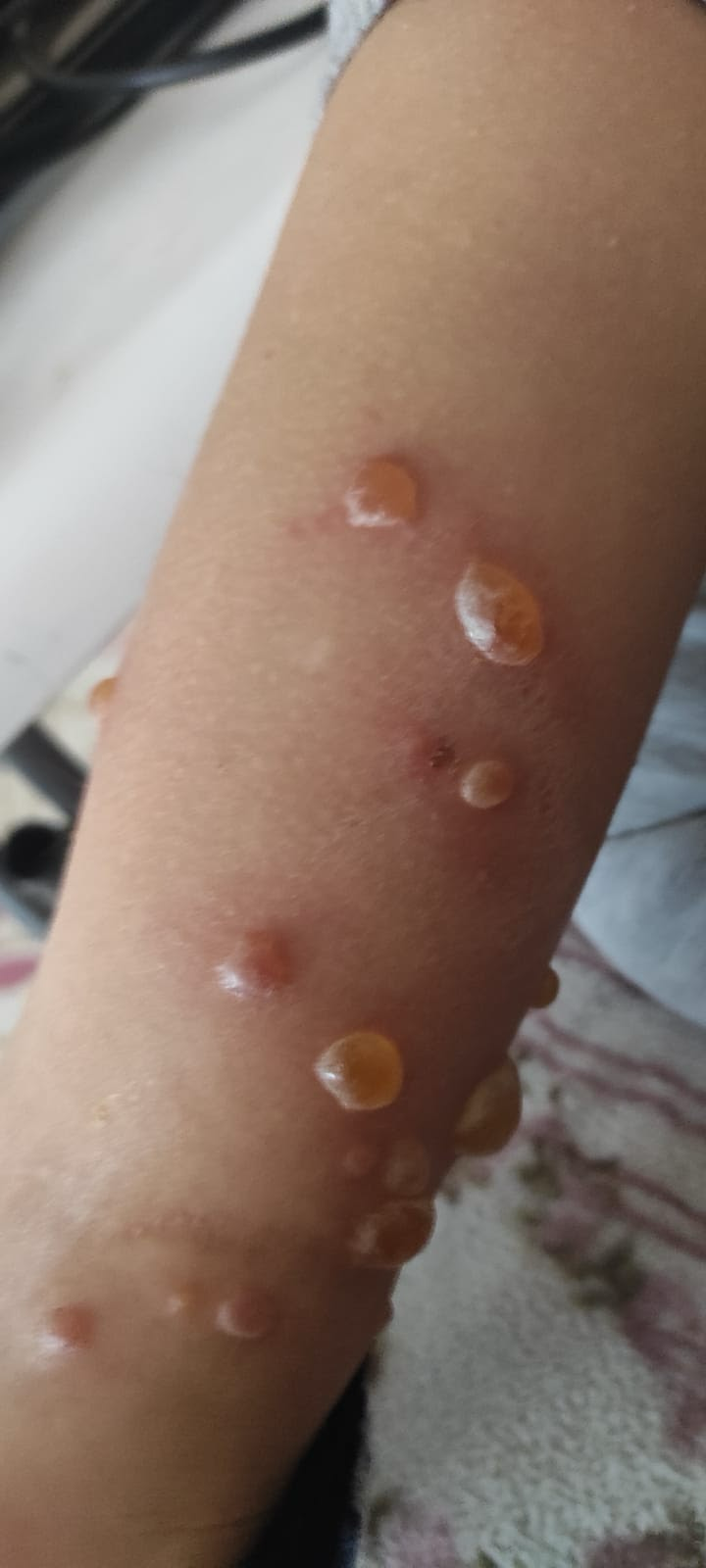


 **Varicella (Chickenpox)**

 **Insect Bite**

 **Herpes Zoster (Shingles)**

 **Molluscum Contagiosum**

 **Bullous Pemphigoid**

43- An **11-month-old male patient** presented with **fever and rash for the past 2 days**. The **rash was itchy**.

**What is your diagnosis?**


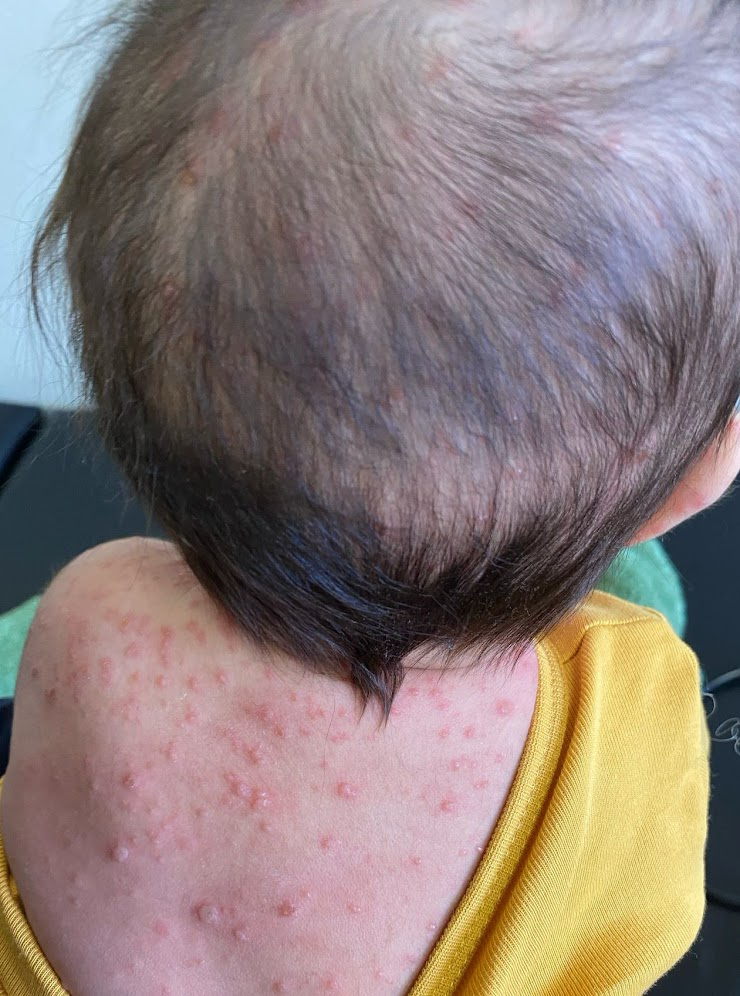


 **Enteroviral Rash (Enteroviral Exanthem)**

 **Measles**

 **Varicella (Chickenpox)**

 **Pityriasis Lichenoides et Varioliformis Acuta (PLEVA)**

 **Insect Bite**

44- An **18-month-old female patient** presented with **itching and rash for the past 4 weeks**. **Similar rashes were present on the hands, feet, and trunk.**

**What is your diagnosis?**


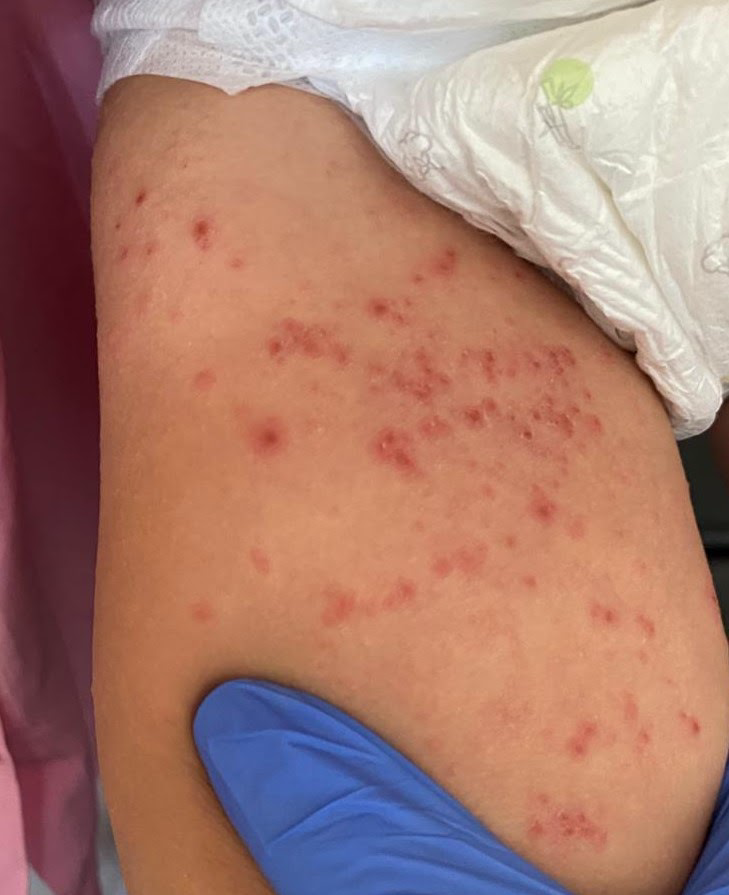


 **Scabies**

 **Enteroviral Rash (Enteroviral Exanthem)**

 **Herpes Zoster (Shingles)**

 **Varicella (Chickenpox)**

 **Pityriasis Lichenoides et Varioliformis Acuta (PLEVA)**

45- A **3-year-old female patient** presented with **itchy skin lesions that started yesterday**. **There were no additional complaints.** The **rash was widespread over the entire body and was migratory (changing location).**

**What is your diagnosis?**


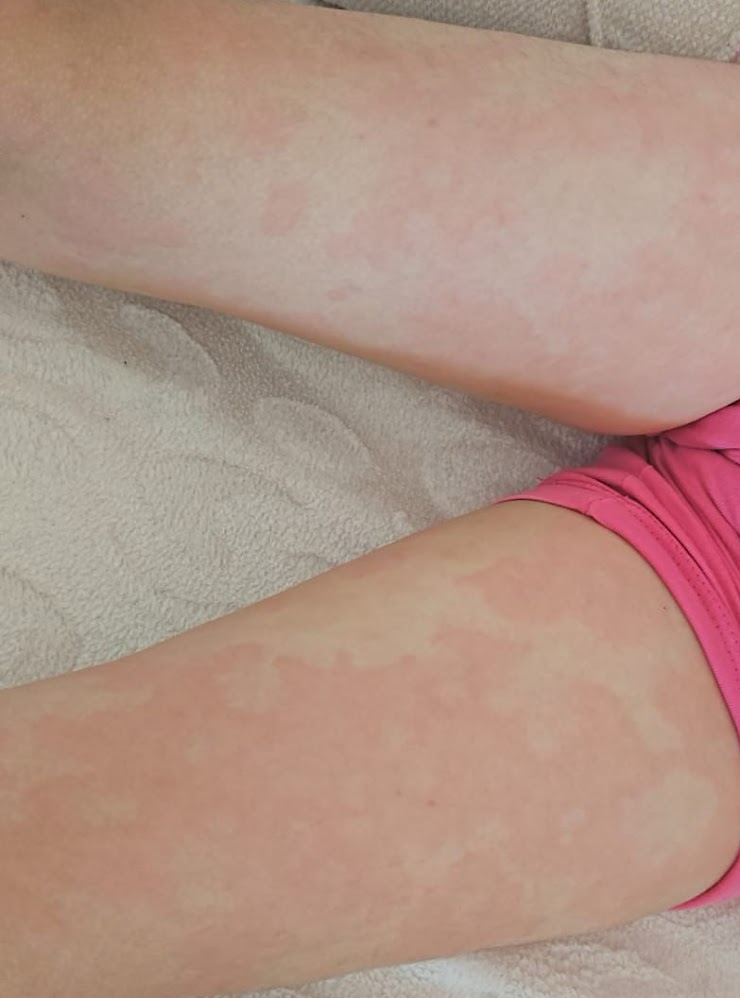


- **Measles**
- **Urticarial Vasculitis**
- **Erythema Marginatum**
- **Urticaria**
- **Cutaneous Mastocytosis**

46- A **2-year-old male patient** had **skin lesions for the past 3 weeks**. **Pruritus (itching) was present.** **No additional findings were noted.**

**What is your diagnosis?**


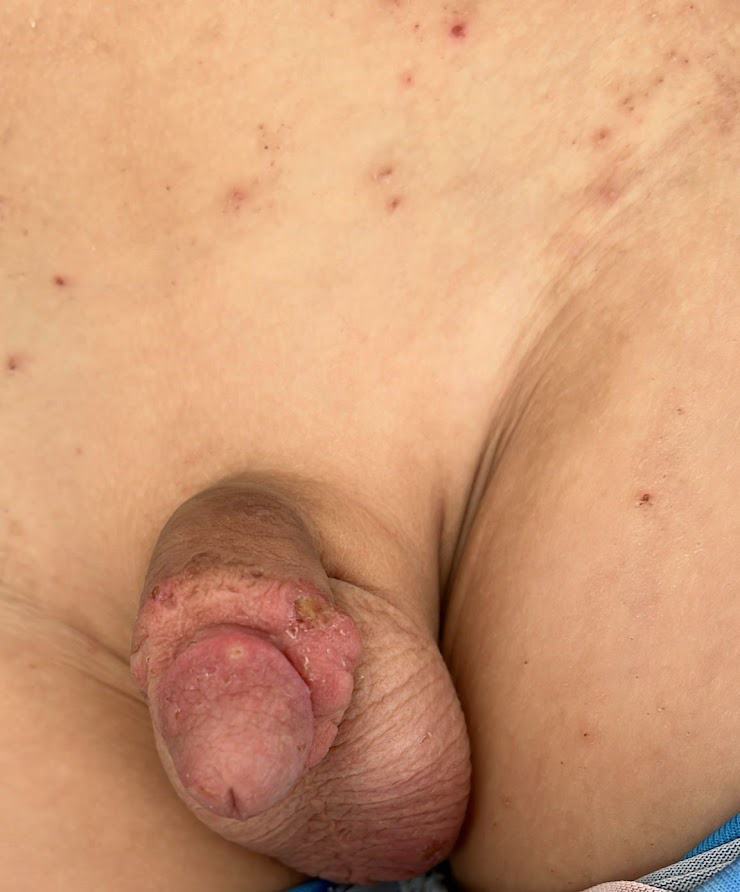


 **Syphilis**

 **Scabies**

 **Enteroviral Rash (Enteroviral Exanthem)**

 **Varicella (Chickenpox)**

 **Pityriasis Lichenoides et Varioliformis Acuta (PLEVA)**

47- A **13-year-old male patient** presented with **painful skin lesions for the past 2 days**. He had **low-grade fever (subfebrile)**.

**What is your diagnosis?**


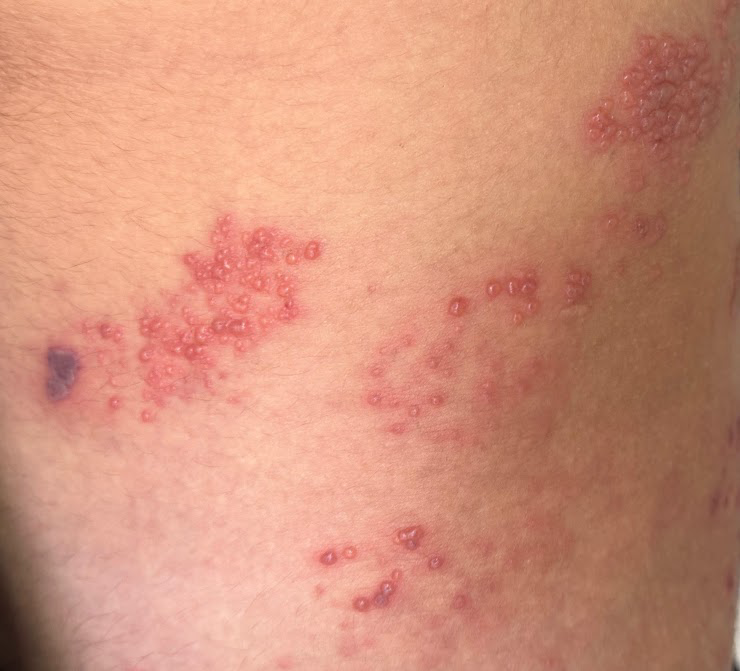


 **Varicella (Chickenpox)**

 **Enteroviral Rash (Enteroviral Exanthem)**

 **Herpes Zoster (Shingles)**

 **Insect Bite**

 **Bullous Pemphigoid**

48- A **12-year-old male patient** presented with **fatigue, low-grade fever, and rash for the past 3 days**.

**What is your diagnosis?**


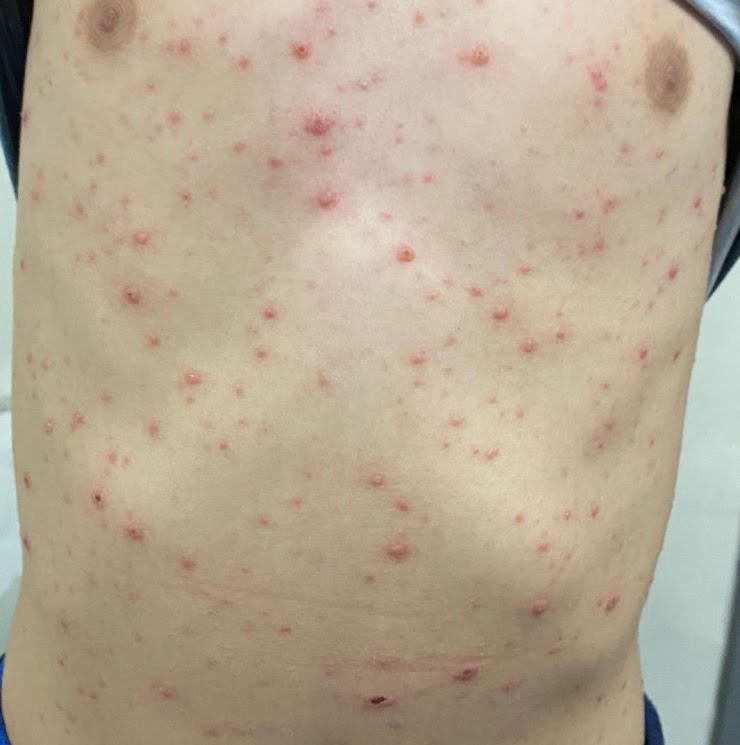


 **Varicella (Chickenpox)**

 **Insect Bite**

 **Bullous Pemphigoid**

 **Measles**

 **Enteroviral Rash (Enteroviral Exanthem)**

49- A **4-month-old female patient** presented with a **rash for the past 3 days**.

**What is your diagnosis?**


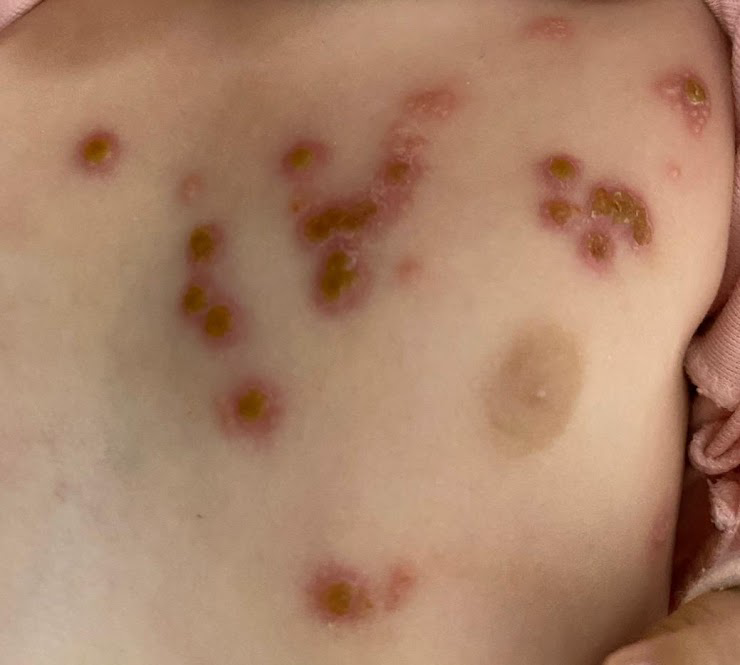


 **Varicella (Chickenpox)**

 **Insect Bite**

 **Impetigo**

 **Scabies**

 **Enteroviral Rash (Enteroviral Exanthem)**

50- A **3-year-old male patient** presented with **skin lesions that have been present for the past 2 months**. **There are no additional complaints.**

**What is your diagnosis?**


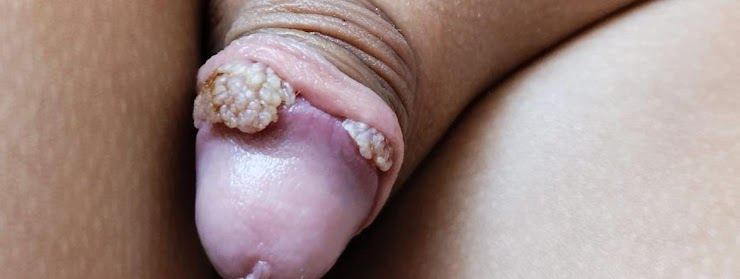


 **Condyloma Acuminata**

 **Molluscum Contagiosum**

 **Basal Cell Carcinoma**

 **Pearly Penile Papules**

 **Seborrheic Keratosis**

51- A **6-year-old female patient** presented with **joint pain, abdominal pain, and rash for the past 2 days**.

**What is your diagnosis?**


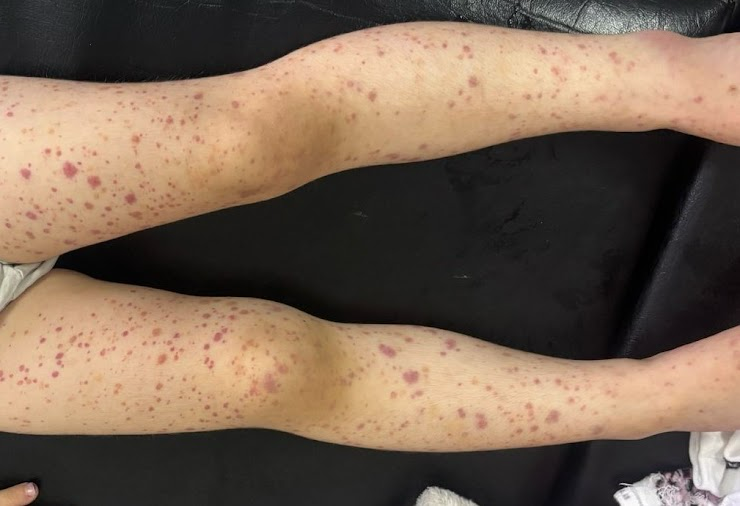


 **Meningococcemia**

 **Henoch–Schönlein Purpura (HSP)**

 **Acute Infantile Hemorrhagic Edema (AIHE)**

 **Drug Rash with Eosinophilia and Systemic Symptoms (DRESS)**

 **Urticarial Vasculitis**

52- A **3-year-old male patient** presented with **facial redness and a lace-like rash on the extremities for the past 2 days**. He had **low-grade fever (subfebrile)**.

**What is your diagnosis?**


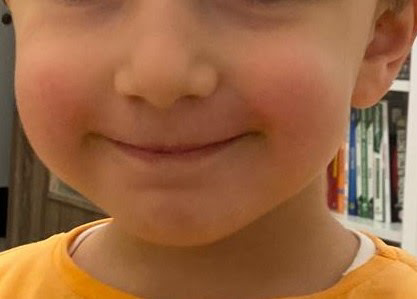


 **Varicella (Chickenpox)**

 **Urticaria**

 **Measles**

 **Parvovirus B19 Infection**

 **Scarlet Fever**

53- A **4-year-old female patient** presented with **lesions on the trunk that have been present for 4 months**.

**What is your diagnosis?**


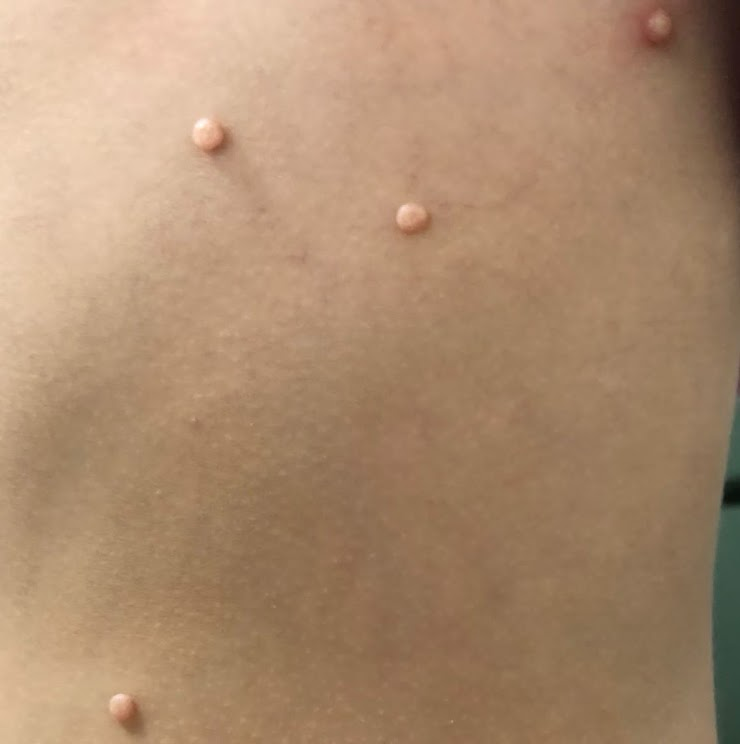


 **Condyloma Acuminata**

 **Molluscum Contagiosum**

 **Basal Cell Carcinoma**

 **Verruca Vulgaris (Common Warts)**

 **Sebaceous Hyperplasia**

54- A **14-month-old male patient** had **persistent fever, cough, and runny nose for the past 3 days**. **There was no fever today**, and the **rash started today**. **Similar rashes were present on the anterior trunk and back**.

**What is your diagnosis?**


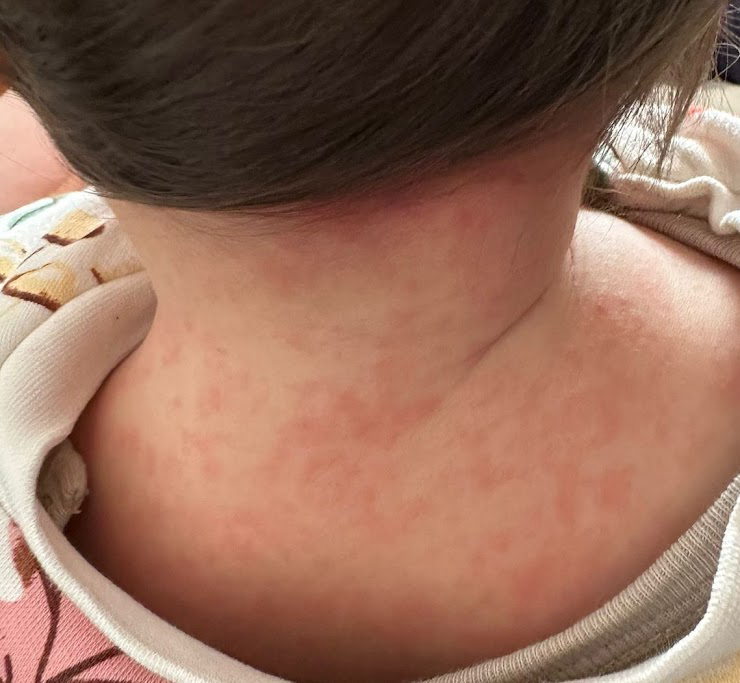


 **Measles**

 **Enteroviral Infection**

 **Sixth Disease (Exanthema Subitum / Roseola)**

 **Parvovirus B19 Infection**

 **Drug Eruption (Drug-related rash)**

55- A **9-year-old male patient** presented with **fever for 3 days, a widespread rash over the entire body, dry and cracked lips, and conjunctivitis**. He has been **taking levetiracetam for epilepsy for the past 1.5 months**.

**What is your diagnosis?**


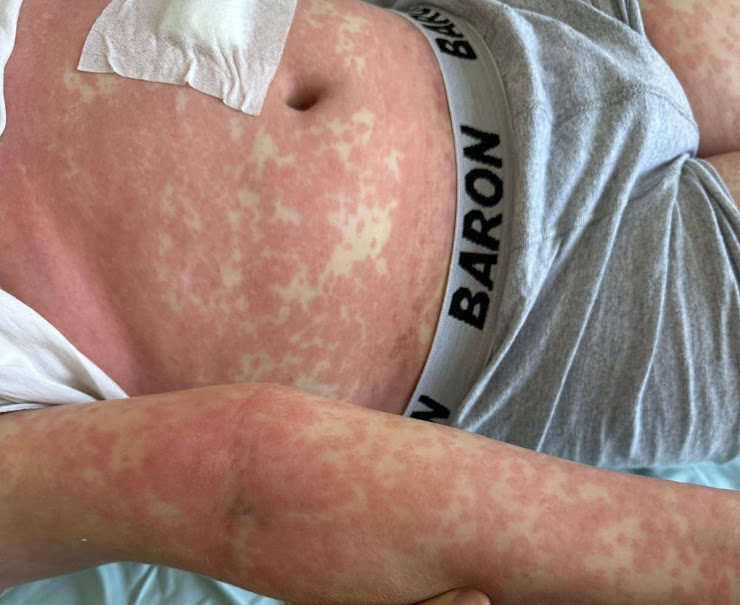


 **Stevens–Johnson Syndrome**

 **Kawasaki Disease**

 **Measles**

 **Urticarial Vasculitis**

 **Henoch–Schönlein Purpura (HSP)**

56- A **7-year-old male patient** presented with **abdominal pain for 3 days, rash for 2 days, and swelling of the ankle**. **What is your diagnosis?**


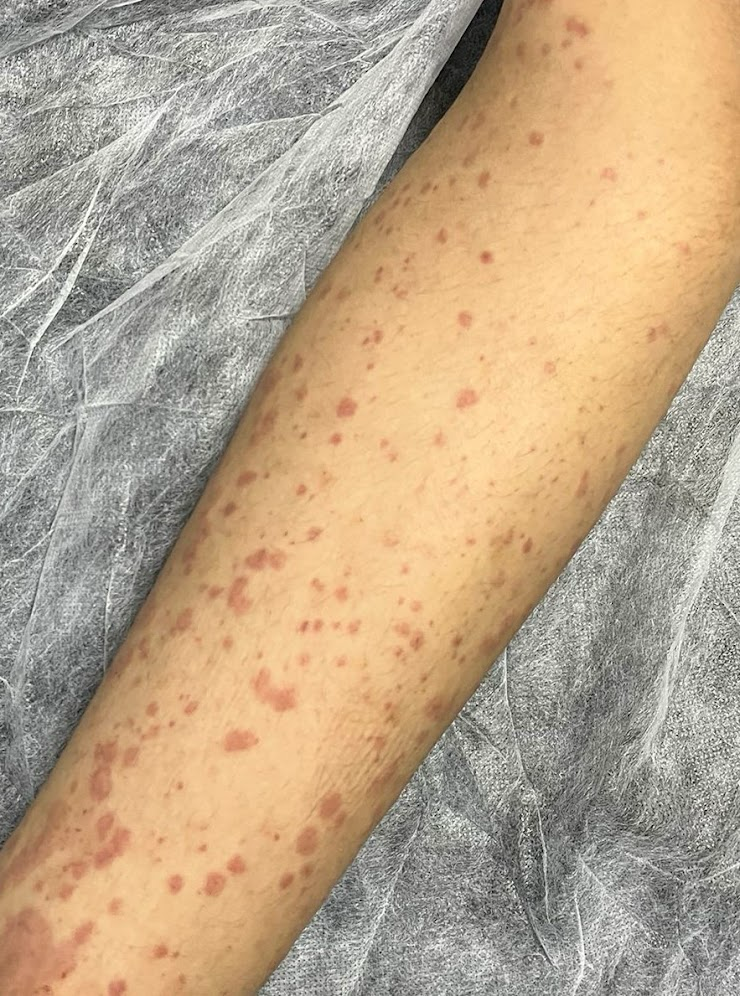


 **Urticarial Vasculitis**

 **Henoch–Schönlein Purpura (HSP)**

 **Acute Infantile Hemorrhagic Edema (AIHE)**

 **Drug Rash with Eosinophilia and Systemic Symptoms (DRESS)**

 **Meningococcemia**

57- 13 A **13-month-old male patient** presented with **fever and similar rashes on the hands and feet for the past 2 days**.

**What is your diagnosis?**


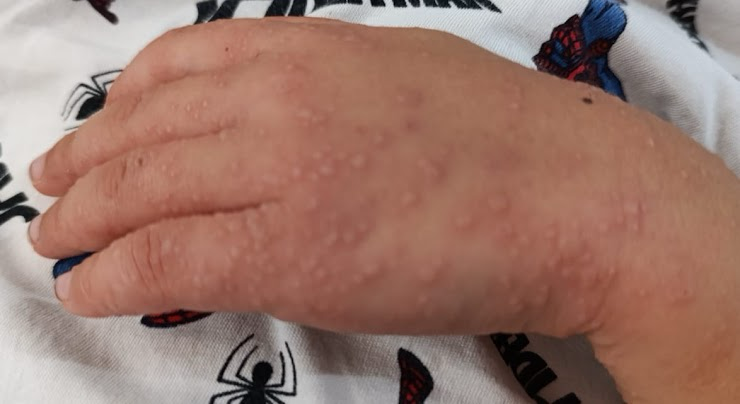


 **Varicella (Chickenpox)**

 **Pityriasis Lichenoides et Varioliformis Acuta (PLEVA)**

 **Scabies**

 **Enteroviral Rash (Enteroviral Exanthem)**

 **Mpox (Monkeypox)**

58- A **10-month-old female patient** presented with a **rash that has been present for 3 weeks**. She had **used antihistamines but did not benefit from them**.

**What is your diagnosis?**


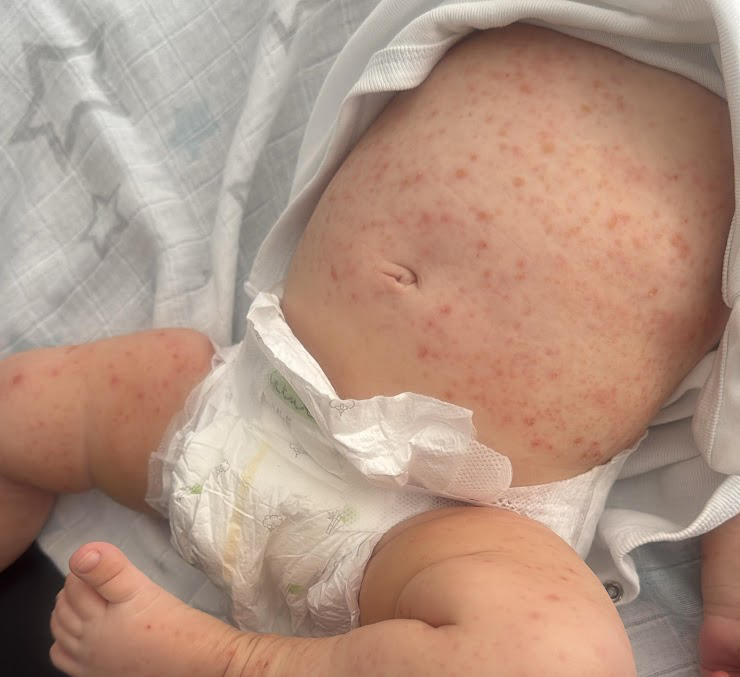


 **Atopic Dermatitis**

 **Scabies**

 **Enteroviral Rash (Enteroviral Exanthem)**

 **Measles**

 **Nummular Dermatitis**

59- A **6-year-old female patient** presented with **fever and pain, swelling, and redness in the area shown in the photograph**.

**What is your diagnosis?**


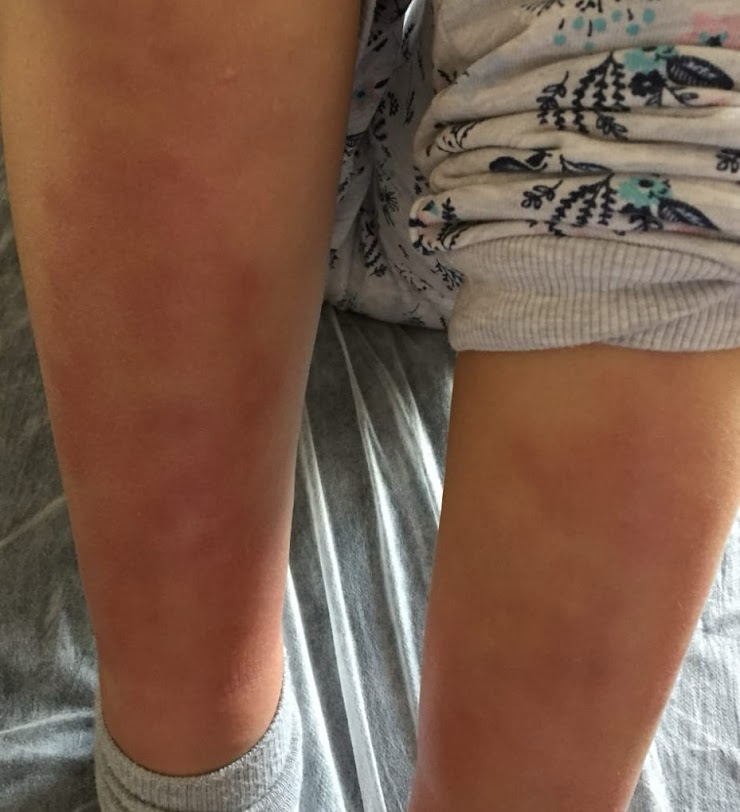


 **Erythema Multiforme**

 **Erythema Nodosum**

 **Subcutaneous Nodule**

 **Anthrax (Cutaneous Anthrax)**

 **Cutaneous Mastocytosis**

60- A **3-year-old female patient** presented with a **rash that has been present for 2 days**. **There were no additional complaints.**

**What is your diagnosis?**


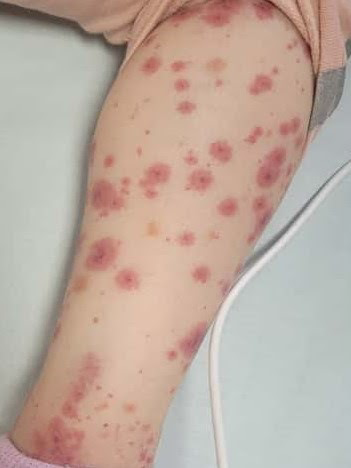


 **Henoch–Schönlein Purpura (HSP)**

 **Meningococcemia**

 **Acute Infantile Hemorrhagic Edema (AIHE)**

 **Disseminated Gonococcal Infection**

 **Drug Rash with Eosinophilia and Systemic Symptoms (DRESS)**

61- An **8-month-old male patient** presented with **low-grade fever and similar rashes over the entire body for the past 2 days**. **What is your diagnosis?**


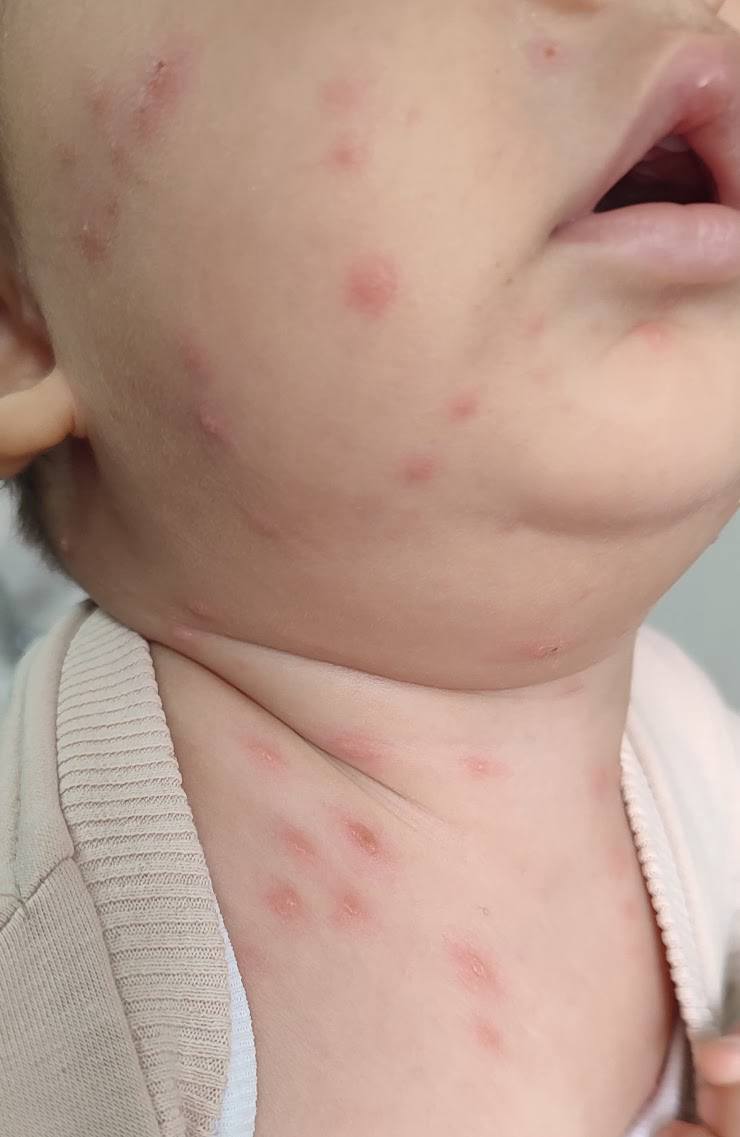


 **Insect Bite**

 **Measles**

 **Enteroviral Rash (Enteroviral Exanthem)**

 **Herpes Zoster (Shingles)**

 **Varicella (Chickenpox)**
